# Design, synthesis, and *in vitro* antiproliferative activity of novel 4-amino-6-phenoxyquinoline and 4,6-diphenoxyquinoline

**DOI:** 10.1039/d6ra01045h

**Published:** 2026-04-30

**Authors:** Ahmed Al-Sheikh, Mohammad Abuoun, Hana'a Khalaf, Nour Albaz, Amna Zaytoun, Duaa Abuarqoub

**Affiliations:** a Department of Pharmacy, Faculty of Pharmacy and Medical Sciences, University of Petra Amman 11196 Jordan aalsheikh@uop.edu.jo Duaa.abuarqoub@uop.edu.jo; b Department of Clinical Nutrition and Diets, Faculty of Pharmacy and Medical Sciences, University of Petra Amman 11196 Jordan

## Abstract

This study aimed to synthesize and biologically evaluate novel 4-amino-6-phenoxyquinoline and 4,6-diphenoxyquinoline derivatives with potential anticancer activity. The newly synthesized compounds were screened for cytotoxicity against a panel of seven human cancer and normal cell lines using the MTT assay to determine IC_50_ values, with sorafenib included as a reference drug. Among the tested molecules, compounds 7a and 7b demonstrated the most potent and selective reduction in cell viability, particularly in A549 lung carcinoma, PANC-1 pancreatic cancer, and MCF-7 breast cancer cells, and were therefore selected for further mechanistic investigations. Flow cytometry-based Annexin V/PI analysis revealed significant induction of apoptosis and necrosis following treatment, with pronounced effects observed in A549 and PANC-1 cells exposed to compound 7a and sorafenib. Acridine orange staining further confirmed a marked increase in autophagic activity across all examined cancer cell lines, indicating the activation of additional cell death pathways. Cell cycle analysis demonstrated compound- and cell-type-dependent effects, including S-phase arrest in A549 and PANC-1 cells and G1-phase arrest in MCF-7 cells. Collectively, these findings indicate that 4-amino-6-phenoxyquinoline derivatives exert anticancer effects through coordinated induction of apoptosis, autophagy, and cell cycle arrest, supporting their potential as promising candidates for further anticancer drug development.

## Abstract 2

This study aimed to synthesize and biologically evaluate novel 4-amino-6-phenoxyquinoline and 4,6-diphenoxyquinoline derivatives with potential anticancer activity.

## Graphical abstract

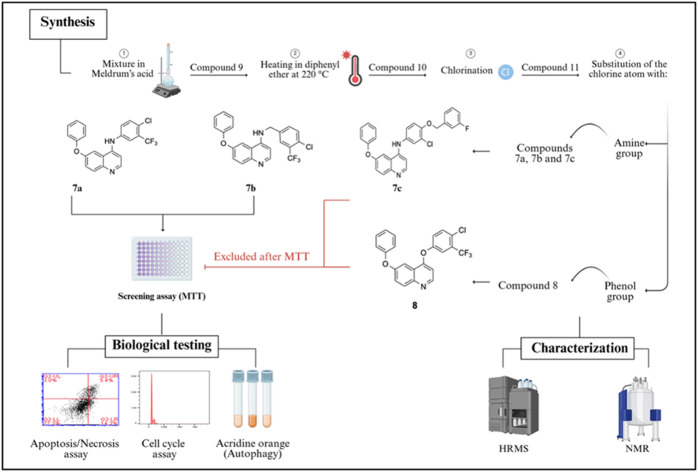

## 1 Introduction

**Fig. 1:**
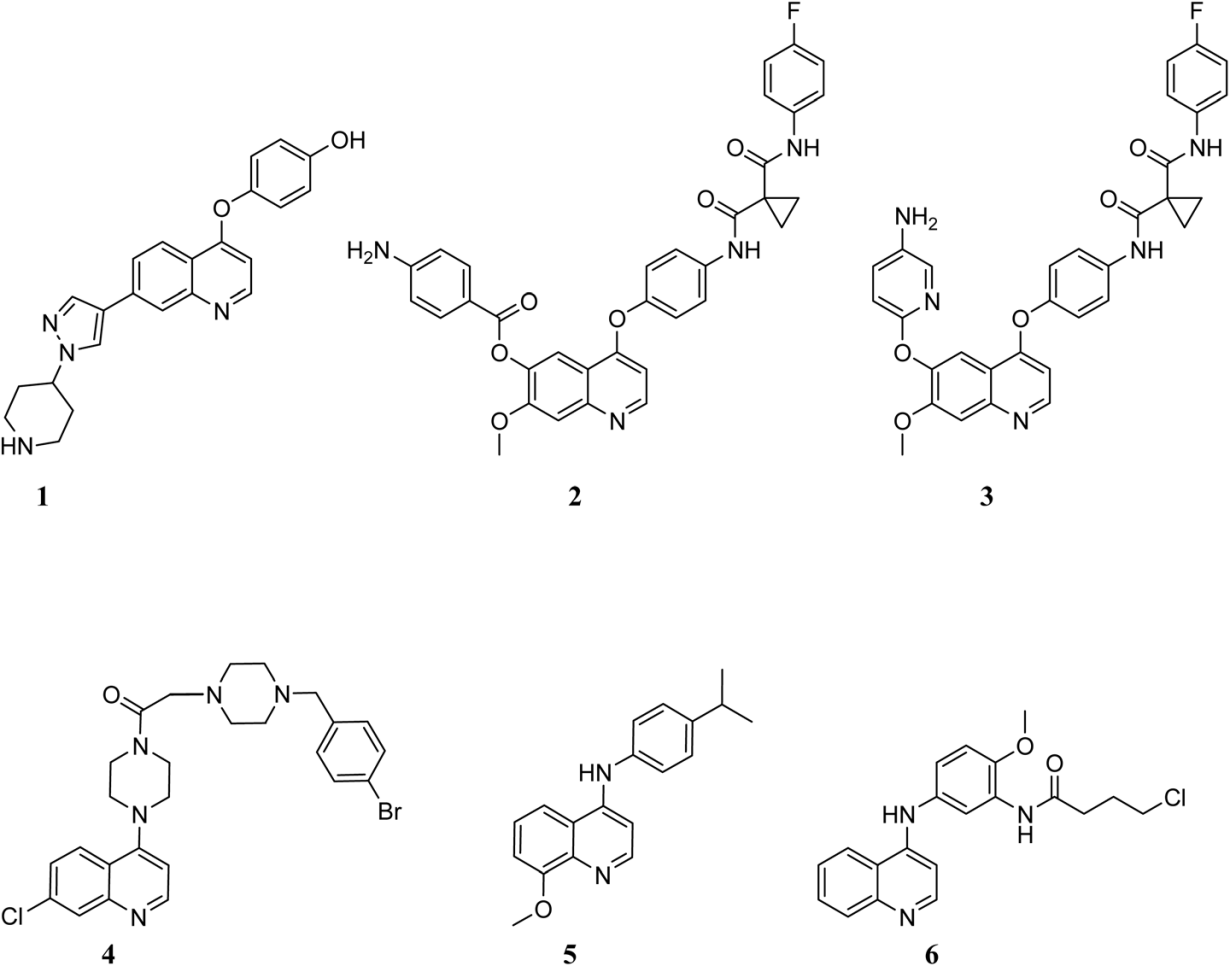
Chemical structures of reported 4-phenoxy and 4-amino quinoline derivatives as anticancer agents.

Cancer is a pathological condition characterized by unregulated cellular proliferation, with the capacity to metastasize or infiltrate adjacent tissues, ultimately resulting in mortality if the spread of cancer cells remains unmitigated. Lung, colorectal, liver, breast, stomach, and pancreatic malignancies are responsible for the highest mortality rates associated with cancer.^1^ Cancer continues to rank as one of the predominant contributors to global mortality. Consequently, the identification and formulation of innovative pharmacological agents capable of eradicating neoplastic cells is of paramount importance. Quinoline derivatives play a pivotal role in oncological therapeutics. In recent years, substantial efforts have been directed towards the discovery of novel small molecules derived from 4-phenoxy and 4-amino quinoline scaffolds, as presented in Fig. 1, that can target specific proteins that are critical for the regulation of cancerous growth and proliferation. Mah *et al.* elucidated examples of 4-phenoxyquinoline derivatives serving as inhibitors of anaplastic lymphoma kinase. The principal derivative 1 demonstrated pronounced antiproliferative properties against H2228 CR cells that exhibit resistance to crizotinib.^2^ Lien *et al.* introduced innovative c-Met inhibitors derived from the cabozantinib framework. Compounds 2 and 3 emerged as the most effective against various cell lines associated with leukemia, the central nervous system, and breast cancer cell lines.^3^ Aboul-Enein *et al.* documented a novel series of 7-chloro-4-(piperazin-1-yl) quinoline derivatives. These compounds underwent *in vitro* anticancer evaluations against human breast cancer (MCF-7) and prostate cancer (PC3) cell lines, while also being assessed for their activity as VEFGR-II inhibitors. The compound 4 exhibited the highest cytotoxic effects against both cell lines.^4^ Liu *et al.* reported a collection of 7-fluoro or 8-methoxy 4-anilinoquinolines. The principal compound 5 demonstrated significant cytotoxic properties against the human cervical cancer cell line (HeLa) as well as the human gastric carcinoma cell line (BGC-823).^5^ Zhou *et al.* documented the synthesis of innovative 4-anilinoquinoline derivatives. The principal compound 6 displayed remarkable antitumor efficacy across various human tumor cell lines (colon: HCT-8, lung: A549, ovarian: A2780S, and breast: MCF-7).^6^

**Fig. 2:**
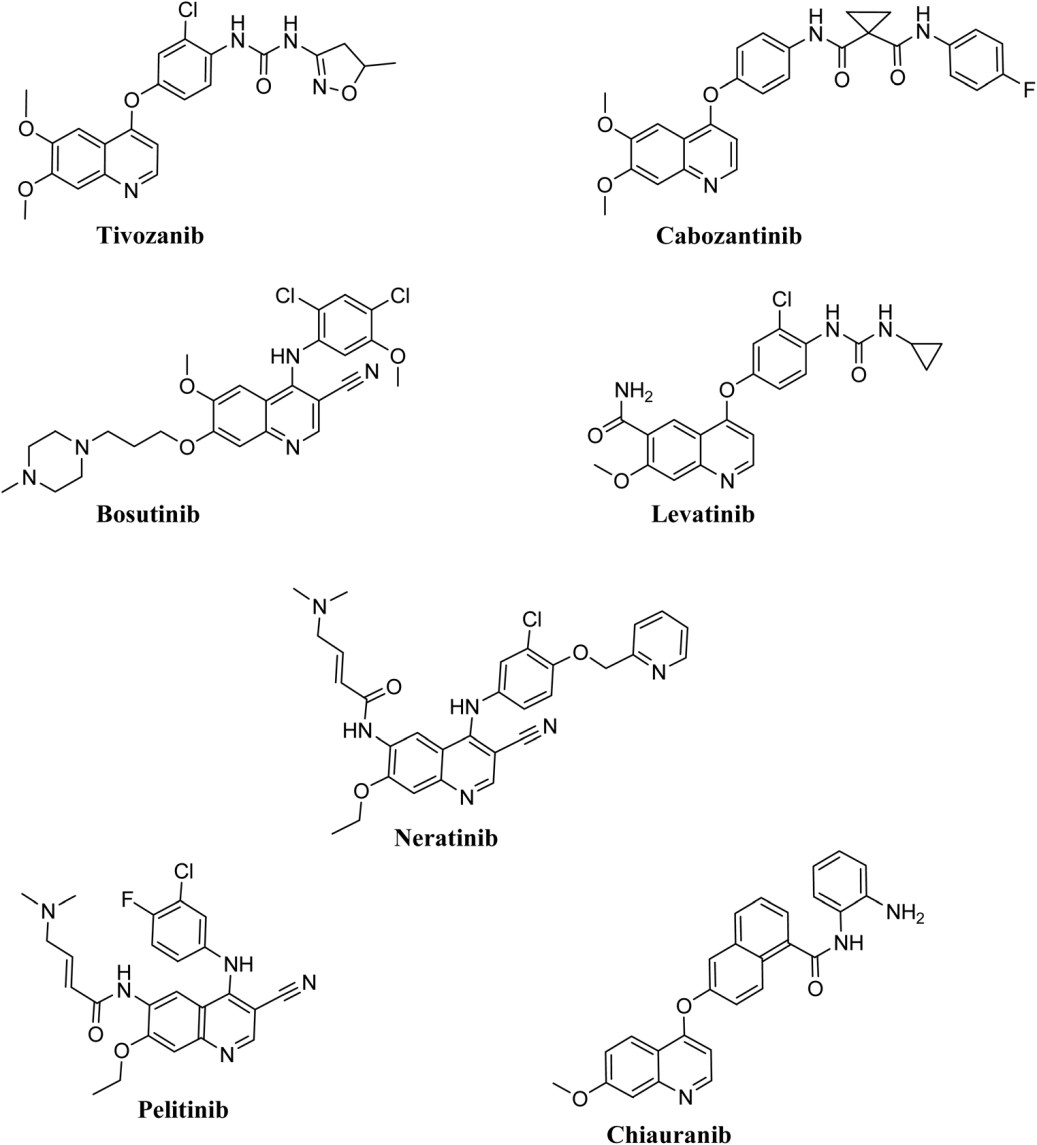
Some of the quinoline-based derivatives are approved for therapy or under clinical trial for the treatment of cancer.

Tivozanib, cabozantinib, bosutinib, lenvatinib, and neratinib represent notable examples of 4-phenoxy and 4-amino quinoline-derived anti-cancer pharmacological agents that have received regulatory approval. Pelitinib and chiauranib, which are also quinoline-based therapeutics, are presently undergoing rigorous evaluation in clinical trials Fig. 2. Tivozanib has been approved for use as a first-line therapeutic option for adult patients diagnosed with advanced renal cell carcinoma (RCC).^7^ Cabozantinib, classified as a tyrosine kinase inhibitor targeting VEGFR, Ret, and Met, has been authorized for the management of metastatic medullary thyroid cancer, advanced renal cell carcinoma, and hepatocellular carcinoma.^8^ Bosutinib, functioning as an inhibitor of Bcr-Abl and Src kinases, has been approved for the treatment of chronic myelogenous leukemia (CML).^9^ Lenvatinib, a receptor tyrosine kinase (RTK) inhibitor that targets VEGFR1 (FLT1), VEGFR2 (KDR), and VEGFR3 (FLT4), has been granted approval for the therapeutic intervention of metastatic thyroid cancer, advanced renal cell carcinoma, and hepatocellular carcinoma.^10^

**Fig. 3:**
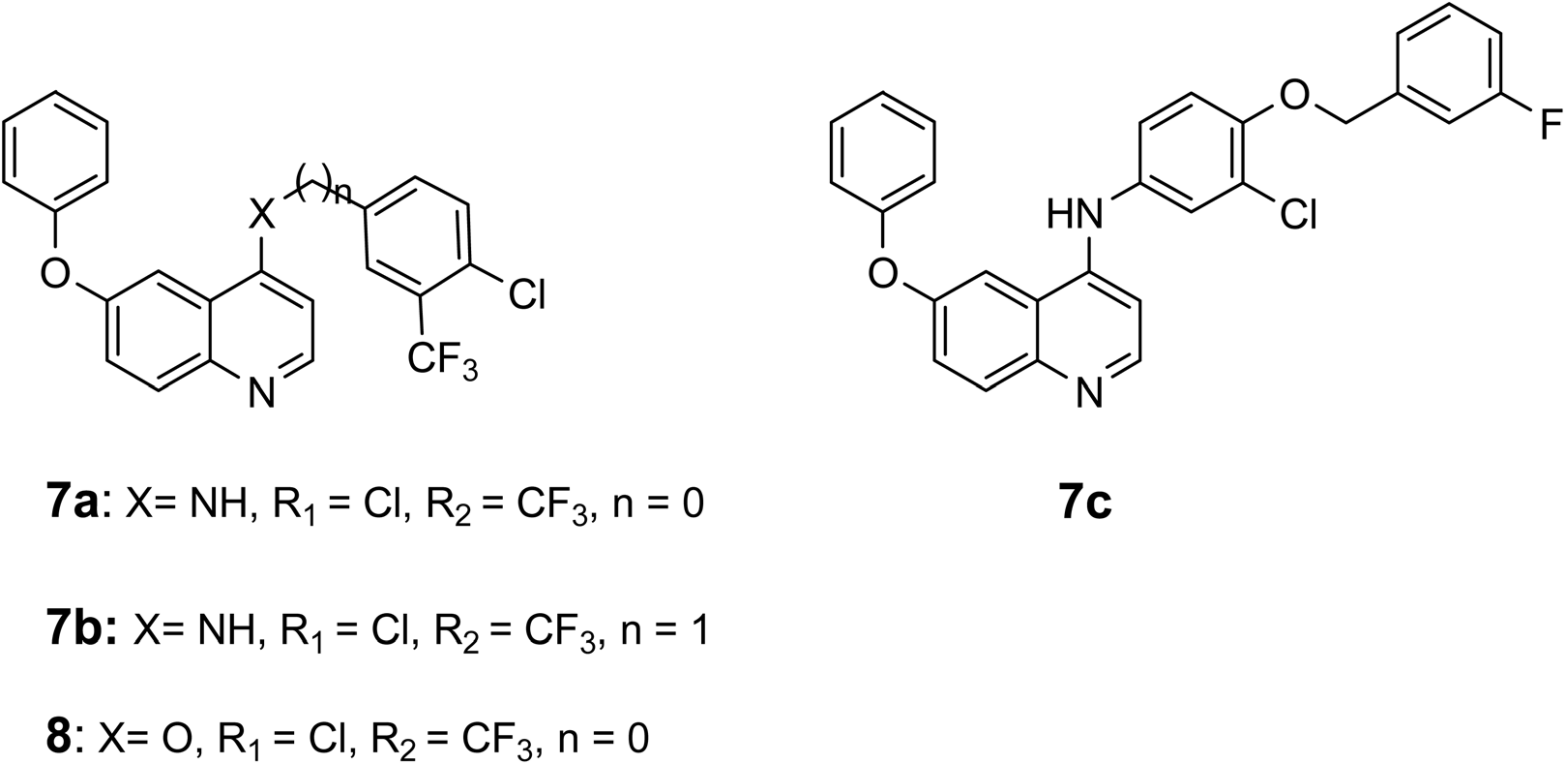
Rational design of 4-amino and 4-phenoxy quinoline derivatives.

From here, and as a continuation of our research endeavors focused on the synthesis of innovative quinoline derivatives for use as anti-cancer agents,^11^ we present the synthesis of the novel 4-amino-6-phenoxyquinoline 7a–7c and 4,6-diphenoxyquinoline 8Fig. 3, alongside their cytotoxic effects on various cell lines, specifically two breast cancer cell lines (MCF-7, MDA-MB-231), glioblastoma (U87), lung cancer (A549), pancreatic cancer (PANC-1), liver cancer (Hep-G2), and normal gingival fibroblast (GF) cells. Furthermore, the underlying mechanism of action was explored through the pathways of apoptosis/necrosis and cell cycle analysis.

## 2 Materials and methods

### 2.1 Synthesis of 4-amino-6-phenoxyquinoline and 4,6-diphenoxyquinoline derivatives

**Scheme 1:**
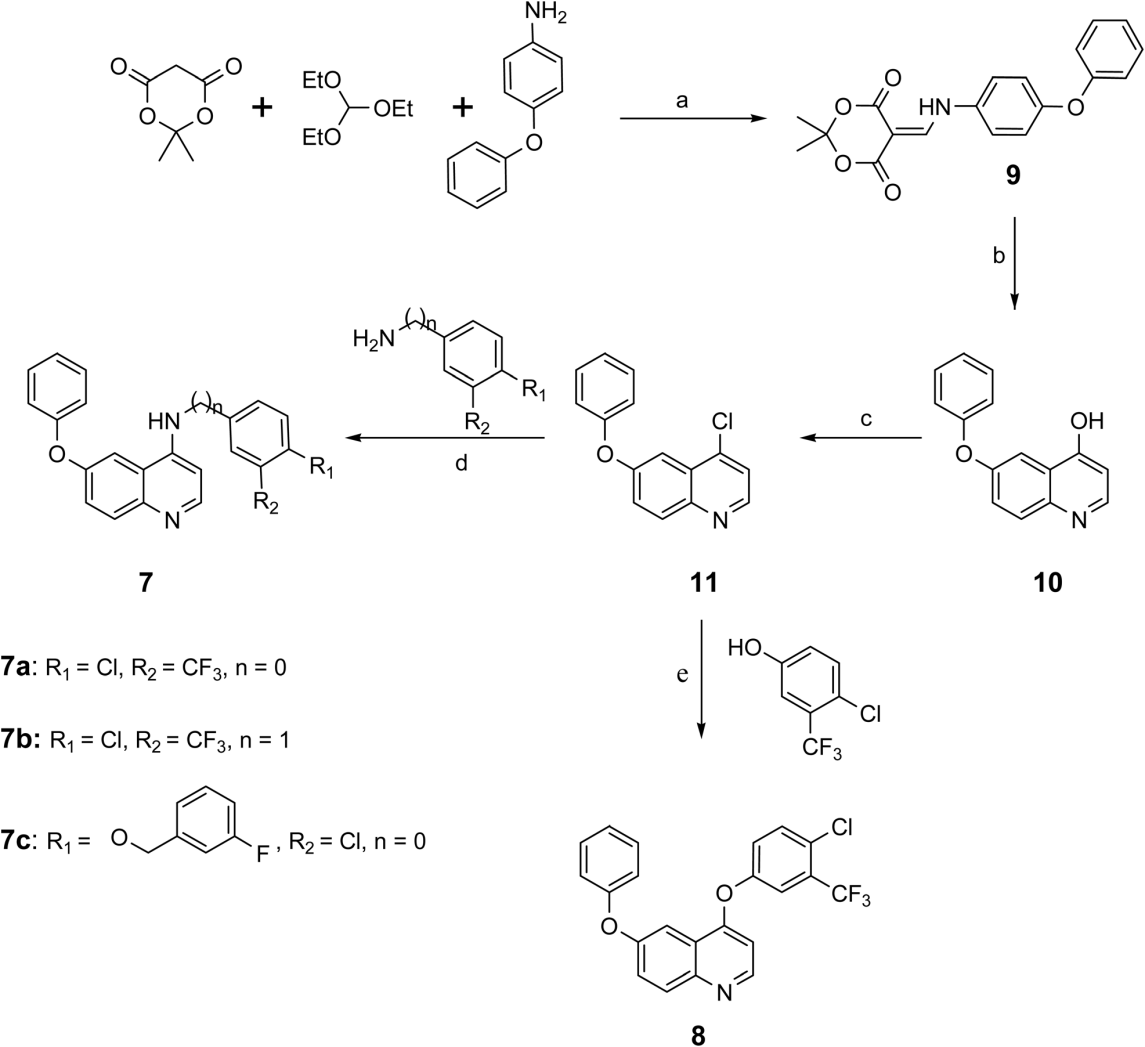
General synthetic pathway for the preparation of 4-amino-6-phenoxyquinoline and 4,6-diphenoxyquinoline derivatives. Reagents and conditions: (a) 85 °C, 1.5 h, 92%; (b) diphenyl ether, 220 °C, 15 min, 43%; (c) phosphorus oxychloride, 95 °C, 3 h, 73%; (d) EtOH, reflux, 24 h, 7a: 91%, 7b: 36%, 7c: 43%; (e) NaOH, 100 °C, 4 h, 52%.

**Fig. 4:**
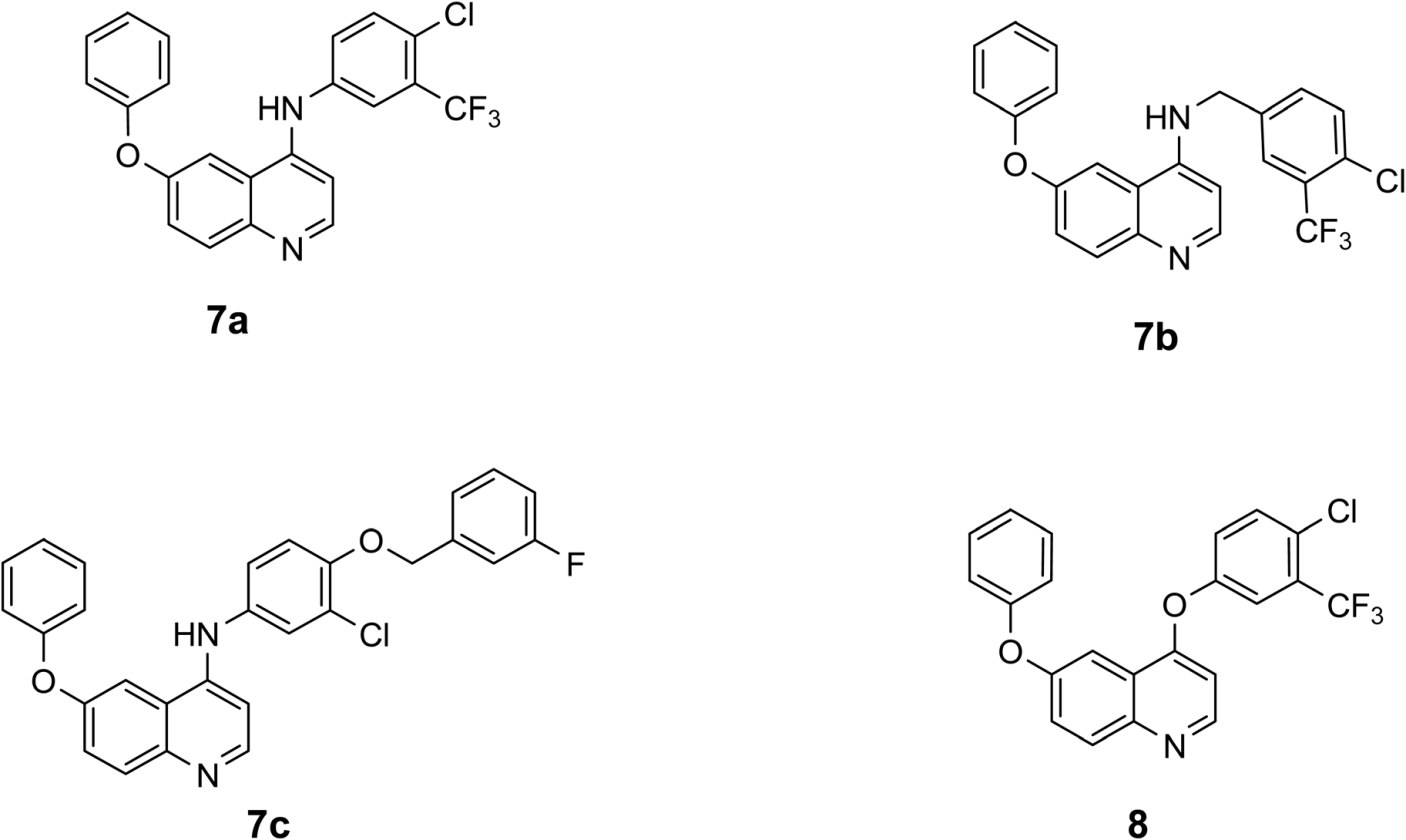
Chemical structures of 4-amino-6-phenoxyquinoline and 4,6-diphenoxy quinoline derivatives.

Three derivatives of 4-amino-6-phenoxyqyinoline 7a–7c and one derivative of 4,6-diphenoxyquinoline 8 were synthesized through the thermal reaction of Meldrum's acid with 4-phenoxyaniline in triethyl orthoformate at a temperature of 85 °C, resulting in the formation of compound 9. The subsequent heating of compound 9 in diphenyl ether at 220 °C facilitated the conversion to 6-phenoxyquinolin-4-ol, designated as compound 10. The chlorination of compound 10 utilizing phosphorus oxychloride led to the formation of 4-chloro-6-phenoxyquinoline, referred to as compound 11. The transformation of compound 11 into derivatives of 4-amino-6-phenoxyquinoline 7a–7c and 4,6-diphenoxyquinoline 8, was achieved through the substitution of the chlorine atom with the respective amine or phenol, as illustrated in Scheme 1 and Fig. 4.

All chemical reagents and solvents were purchased from commercial sources and used without additional purification processes. All nuclear magnetic resonance (NMR) spectra were recorded in deuterated DMSO utilizing a Bruker spectrometer operating at a frequency of 500 MHz. High-Resolution Mass Spectra (HRMS) were generated using an electrospray ionization technique on a Bruker Impact II Mass Spectrometer in positive ionization mode at a voltage of 2500 V. The comprehensive synthetic protocols for the preparation of the novel 4-amino-6-phenoxyquinoline derivatives, as outlined in Scheme 1, are detailed in the following subsections. (Fig S1)

#### 2.1.1 Compound 9 (2,2-dimethyl-5-[(4-phenoxyanilino)methylidene]-1,3-dioxane-4,6-dione)

In a solution of Meldrum's acid (2 g, 13.89 mmol) in triethyl orthoformate (15 mL), 4-phenoxyaniline (5.14 g, 27.77 mmol) was incorporated. The reaction mixture was subjected to heating at 85 °C for a duration of 1.5 hours. Subsequent to the cooling of the mixture to ambient temperature, the precipitate formed was subjected to filtration and subsequently washed with diethyl ether, resulting in the isolation of compound 9 as a light-yellow solid (4.23 g, 92%). The isolated intermediate was utilized in the subsequent synthetic step without undergoing any additional purification processes.

#### 2.1.2 Compound 10 (6-phenoxyquinolin-4-ol)

A 10 g of diphenyl ether was subjected to heat at a temperature of 220 °C. Subsequently, 1 g of compound 9 (2.95 mmol) was introduced into the reaction mixture. The resultant solution was stirred at a temperature of 220 °C for a 15 minute duration. The resultant precipitate was subsequently isolated through filtration and was washed with diethyl ether, yielding compound 10 as a light-yellow solid (0.3 g, 43%). The isolated intermediate was employed in the subsequent step without undergoing any further purification.

#### 2.1.3 Compound 11 (4-chloro-6-phenoxyquinoline)

A solution composed of 0.66 g of compound 10 (6-phenoxyquinolin-4-ol) (2.78 mmol) in 2 mL of phosphorus oxychloride was subjected to heating at a temperature of 95 °C for 3 hours. After cooling to room temperature, the resultant mixture was transferred onto 50 mL of crushed ice. The solution was then cooled to 0 °C and neutralized through the gradual addition of a saturated sodium bicarbonate (NaHCO_3_) solution. The mixture underwent extraction utilizing 25 mL of 2× dichloromethane. The integrated organic layers were subjected to drying over sodium sulfate (Na_2_SO_4_), followed by filtration and concentration under vacuum, ultimately resulting in the formation of 0.52 g of compound 11 as a light-yellow solid (73%).^1^H NMR (500 MHz, CDCl_3_) *δ*: 8.69–7.11 (m, 10H). ^13^C NMR (126 MHz, CDCl_3_) *δ*: 156.73, 156.18, 148.28, 145.81, 141.57, 131.83, 130.10, 127.61, 124.36, 123.98, 121.60, 119.63, 109.57. HRMS (ESI, ([M + H]^+^): calculated 256.0523; found, 256.0513.

#### 2.1.4 Compounds 7a, 7b, and 7c (4-amino-6-phenoxyquinoline)

To a solution of 0.15 g of compound 11 (0.58 mmol, 1 equivalent) in 5 mL of ethanol, the corresponding amine (0.65 mmol, 1.1 equivalents) was introduced. The resultant mixture was subjected to reflux overnight. Subsequently, the ethanol was evaporated under vacuum. The resulting residue was washed with diethyl ether and then subjected to filtration to obtain compounds 7a, 4b, and 7c. The following is the structural characterization of compounds 7a, 7b, and 7c.

##### 2.1.4.1 Compound 7a (*N*-[4-chloro-3-(trifluoromethyl)phenyl]-6-phenoxyquinolin-4-amine)

Yield: (0.22 g, 91%).^1^H NMR (500 MHz, CDCl_3_) *δ*: 11.17 (s, 1H), 8.94–6.55 (m, 13H).^13^C NMR (126 MHz, CDCl_3_) *δ*: 156.08, 155.83, 154.53, 140.16, 136.26, 134.95, 132.89, 130.56, 130.06, 129.90, 129.69, 129.44, 125.85, 124.52, 124.44, 123.28, 122.59, 121.11, 119.43, 118.99, 112.67, 99.18. HRMS (ESI, ([M + H]^+^): calculated 415.0819; found, 415.0834.

##### 2.1.4.2 Compound 7b (*N*-{[3-chloro-4-(trifluoromethyl)phenyl]methyl}-6-phenoxyquinolin-4-amine)

Yield: (0.09, 36%).^1^H NMR (500 MHz, CDCl_3_) *δ*: 8.76–6.33 (m, 13H), 4.68 (s, 1H), 4.04 (s, 2H). ^13^C NMR (126 MHz, CDCl_3_) *δ*: 152.02, 149.75, 148.06, 139.47, 134.06, 131.83, 131.68, 128.44, 126.98, 126.90, 126.79, 126.61, 126.32, 125.16, 122.94, 121.48, 119.65, 118.90, 116.77, 114.44, 113.64, 106.02, 93.48, 41.10. HRMS (ESI, ([M + H]^+^): calculated 429.0976; found, 429.0940.

##### 2.1.4.3 Compound 7c (*N*-[(3-chloro-4-[(3-fluorophenyl)methoxy]-6-phenoxyquinolin-4-amine)

Yield: (0.12 g, 43%).^1^H NMR (500 MHz, CDCl_3_) *δ*: 10.88 (s, 1H), 8.91–6.44 (m, 17H), 5.09 (s, 2H). ^13^C NMR (126 MHz, CDCl_3_) *δ*: 163.96, 161.99, 156.02, 155.73, 155.07, 153.10, 139.77, 138.59, 138.53, 134.81, 130.61, 130.37, 130.30, 130.01, 127.80, 125.62, 125.42, 124.26, 123.91, 122.45, 118.93, 115.17, 115.00, 114.29, 113.98, 113.80, 112.70, 98.98, 58.35. HRMS (ESI, ([M + H]^+^): calculated 471.1270; found, 471.1257.

#### 2.1.5 Compound 8 (4-[4-chloro-3-(trifluoromethyl)phenoxy]-6-phenoxyquinoline)

4-chloro-3-(trifluoromethyl)phenol (1.14 g, 5.8 mmol) and NaOH (0.078 g, 1.9 mmol) were heated to 100 °C with stirring until all NaOH pellets were dissolved. 4-chloro-6-phenoxyquinoline 11 (0.15 g, 0.58 mmol) was added. The resulting mixture was stirred at 100 °C for 4 h. After cooling, the mixture was diluted with 10% aq. NaOH (5 mL) and stirred at room temperature for 1 h. The aqueous phase was extracted with dichloromethane (2 × 25 mL). The combined organic layers were washed with water (20 mL), dried over Na_2_SO_4_ and filtered. The dichloromethane was evaporated under vacuum. The resulting residue was washed with diethyl ether and filtrated to yield 8 as a white solid (0.126 g, 52%). ^1^H NMR (500 MHz, CDCl_3_) *δ*: 8.67–6.58 (m, 13H). ^13^C NMR (126 MHz, CDCl_3_) *δ*: 159.95, 156.45, 155.93, 153.06, 149.53, 146.83, 133.27, 131.39, 130.42, 130.16, 130.04, 128.64, 125.00, 124.13, 124.12, 123.25, 122.08, 121.07, 120.30, 120.25, 120.21, 119.51, 107.16, 105.38, 77.30, 77.05, 76.79. HRMS (ESI, ([M + H]^+^): calculated 416.0659; found, 416.0612.

### 2.2 Cell culture

4-amino-6-phenoxyquinoline derivatives were evaluated across seven distinct cell lines in the present study. This study included two breast cancer cell lines (MCF-7, MDA-MB-231), a glioblastoma cell line (U87), a lung cancer cell line (A549), a pancreatic cancer cell line (PANC-1), a liver cancer cell line (Hep-G2), and normal gingival fibroblast (GF) cells. U87 and Hep-G2 cells were seeded in high-glucose DMEM (Euroclone, Italy) media, whereas MCF-7, A549, and PANC-1 were cultured in RPMI-1640 (Euroclone, Italy) media, MDA-MB-231 was seeded in MEM (Euroclone, Italy) media, and GF cells were cultured in α-MEM (Gibco, USA) media. All cancer cell lines were purchased from ATCC, USA. GF cells were donated from the stem cell research lab at the University of Petra Pharmaceutical Center (UPPC). All types of culture media were supplemented with 10% FBS, 1% l-glutamine (Euroclone, Italy), 1% penicillin (Euroclone, Italy), and 0.1% amphotericin B (Euroclone, Italy). Cells were cultured in 75T flasks within a humidified incubator maintained at 5% CO_2_ and 37 °C. Cells were passaged upon reaching 90% confluency utilizing trypsin–EDTA (Euroclone, Italy).

### 2.3 Viability assay

To evaluate the impact of compound 11, compound 7a, compound 7b, and compound 7c derivatives, along with sorafenib as a positive control, on the viability of various cell lines, the (3-(4,5-dimethyl thiazol-2yl)-2,5-diphenyltetrazolium bromide) MTT assay (Promega, Madison, WI, USA) was performed in accordance with the manufacturer's specifications. Briefly, 5 × 10^3^ cells were seeded into each well of a 96-well plate (SPL Life Sciences Co., Ltd, Gyeonggi-do, Korea) and subsequently incubated for 24 hours within a CO_2_ incubator to facilitate optimal cell attachment prior to treatment exposure. Each cell line was then subjected to two distinct concentrations of each pharmacological compound; specifically, 3 µM and 30 µM, for a duration of 72 hours. Following this exposure, the media containing the treatments were substituted with 100 µl of fresh medium containing 15 µl of MTT reagent, and the cells were incubated at 37 °C for a period of 3 hours. Subsequently, the resulting formazan crystals were solubilized by the addition of 50 µl of stop solution. The optical density (OD) was quantified at 570 nm utilizing a plate reader (Thermofisher, USA).

To determine the percentage of cell viability, the following formula was employed: viability percentage = (sample OD/the average of OD of negative control) × 100. Based on the viability results, the most pronounced decrease in viability by the derivatives will be used as a treatment for further investigation on the cell lines, in addition to normal GF cells, utilizing a broader range of concentrations commencing from 50 µM and a gradual 2-fold serial dilution of concentrations until reaching 1.25 µM. To calculate the half maximal inhibitory concentrations (IC_50_) for each derivative, non-linear regression analysis of the logarithmic concentration against the percentage of inhibition was conducted utilizing GraphPad Prism.

### 2.4 Apoptosis/necrosis assay

The modalities of cell death were evaluated utilizing the Annexin V/propidium iodide (PI) detection kit (Abcam, UK). In summary, 3 × 10^5^ PANC-1, MCF-7, A549, and GF cell lines were seeded in six-well plates (SPL Life Sciences Co., Ltd, Gyeonggi-do, Korea) for a duration of 24 hours. Subsequently, the cells were treated with the IC_50_ concentration as well as double the IC_50_ concentration of compound 7a, compound 7b, and sorafenib, as summarized in Table 1, and were subsequently incubated for 72 hours within a CO_2_ incubator. Cells that received treatment with sorafenib were designated as the positive control, whereas those that were solely cultured in media were classified as the negative control.

**Table 1:** The half maximal inhibitory concentrations (IC_50_) and double the IC_50_ values of compound 7a, compound 7b, and sorafenib on PANC-1, MCF-7, and A549 cell lines

	Compound 7a	Compound 7b	Sorafenib
PANC-1	28 µM, 56 µM	11 µM, 22 µM	13 µM, 26 µM
MCF-7	25 µM,50 µM	10 µM,20 µM	12 µM,24 µM
A549	25 µM,50 µM	11 µM,22 µM	20 µM,40 µM

Upon completion of the incubation period, cells were collected through trypsinization and subsequent centrifugation to facilitate staining with annexin V/PI reagents in accordance with the protocols provided by the manufacturer. Following the staining procedure, the samples were analyzed by a flow cytometer (BD Accuri C6, USA), and the resultant data were interpreted employing the Flowjo software (Flowjo, BD Biosciences).

### 2.5 Acridine orange staining (autophagy)

Acridine orange staining was used to examine the formation of phagosomes within cells subsequent to treatment with the 4-amino-6-phenoxyquinoline derivatives 7a and 7b. In brief, 120 × 10^3^ cells of PANC-1, MCF-7, A549, and GF were seeded in six-well plates and incubated for a duration of 24 hours in a CO_2_ incubator. Following this incubation period, the cells underwent treatment for 72 hours utilizing the IC_50_ concentration along with double the amount of compound 7a, compound 7b, and sorafenib, as presented in Table 1. Subsequently, cells were collected through trypsinization and subjected to staining with 10 µM of acridine orange stain (Sigma Aldrich, USA) for a duration of 15 minutes in a dark environment, after which the cells were washed with 1 mL of PBS. The samples were analyzed by flow cytometry (BD Accuri, USA), and the data were processed using Accuri C6 software.

### 2.6 Cell cycle analysis

The progression of the cell cycle was conducted by using propidium iodide (PI) (Tocris, USA) staining to quantify the DNA content of the cells, followed by analysis *via* flow cytometry. In summary, 3 × 10^5^ cells from the PANC-1, MCF-7, A549, and GF cell lines were plated in six-well plates and incubated for a duration of 24 hours within a CO_2_ incubator. Subsequently, the cells underwent treatment for a period of 72 hours with the IC_50_ concentration, as well as double the concentration of compound 7a, compound 7b, and sorafenib, as shown in Table 1. Following the trypsinization and centrifugation of the cells, the supernatant was eliminated, and ice-cold ethanol was added to the cells for 30 minutes; subsequently, the cells were washed twice with ice-cold PBS through centrifugation at 300×*g* for 5 minutes for each wash cycle. Thereafter, the cells were incubated with 200 µl of 20 µg mL^−1^ RNase (NZTtech, Portugal) and 40 µg mL^−1^ PI in PBS at a temperature of 4 °C in darkness. Subsequently, 200 µl of PBS was added to the stained cells, and the samples were analyzed *via* a flow cytometer, and the data were processed using Flowjo software.

### 2.7 Statistical analysis

All experiments and measurements that were performed using flow cytometry were in three replicates, while MTT assay were in four replicates. The results were expressed as mean ± standard deviation (SD) in each experiment. One-way analysis of variance (ANOVA), followed by Dunnett's test for multiple group comparison, was used to determine the statistical difference in the MTT assay and acridine orange staining. Two-way ANOVA was used to perform multiple group comparisons in apoptosis/necrosis and cell cycle assays. All data were analyzed using GraphPad Prism 8.0.1. (GraphPad, USA) and/or Microsoft Excel 16.98 (Microsoft, USA).

## 3 Results

### 3.1 Viability assay (MTT)

**Fig. 5:**
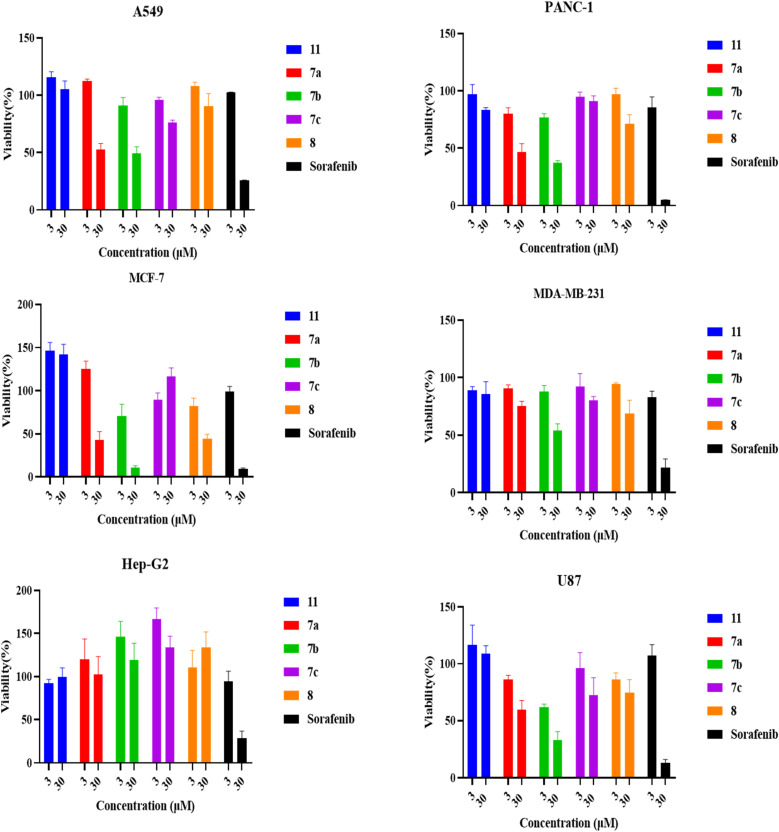
MTT assay performed on a panel of human cancer cell lines (U87, Hep-G2, MCF-7, MDA-MB-231, A549, and PANC-1) treated with 3 and 30 µM (11, 7a, 7b, 7c, 8), in addition to Sorafenib as a positive control. Cell viability was normalized to the negative untreated control.

**Fig. 6:**
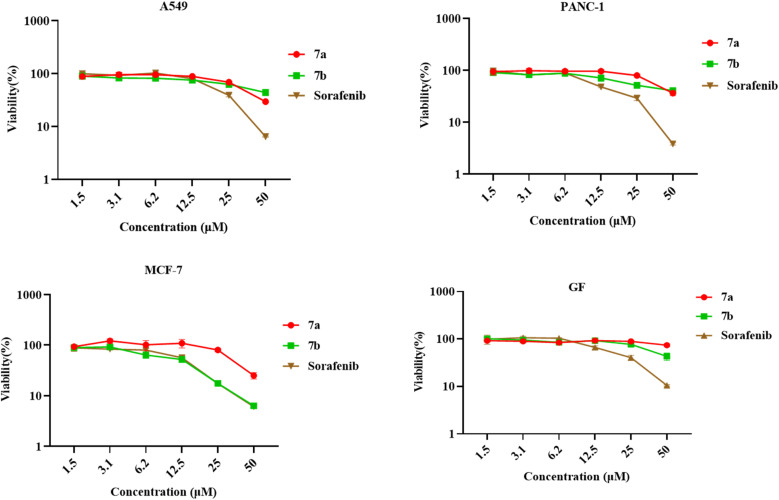
Half-maximal inhibitory concentration (IC_50_) curve of different concentrations (1.5, 3.1, 6.2, 12.5, 25, and 50 µM) of compound 7a, 7b, and Sorafenib on A549, PANC-1, MCF-7, and GF cell lines after 72 hours of treatment. Values are expressed in four replicates as mean ± standard deviation (SD). IC_50_ was calculated by normalizing and log 10-transforming the percentages of cell viability relative to the untreated control, and then nonlinear regression was performed.

The cytotoxic effects of 6-chloroquinoline 11, 4-amino-6-phenoxyquinoline 7a–7c and 4,6-diphenoxyquinoline 8 derivatives on the proliferation of various cell lines were assessed utilizing the MTT assay. A profound reduction in cell viability was noted when A549, PANC-1, and MCF-7 cell lines were subjected to treatment with compounds 11, 7a, 7b, 7c and 8, as illustrated in Fig. 5. Consequently, as mentioned before, additional investigations were done employing an expanded range of concentrations (1.5, 3.1, 6.2, 12.5, 25, and 50 µM) of the aforementioned compounds 7a and 7b on the A549, PANC-1, MCF-7, and GF cell lines, to calculate the IC_50_ values as shown in Fig. 6.

### 3.2 Apoptosis/necrosis assay

**Fig. 7:**
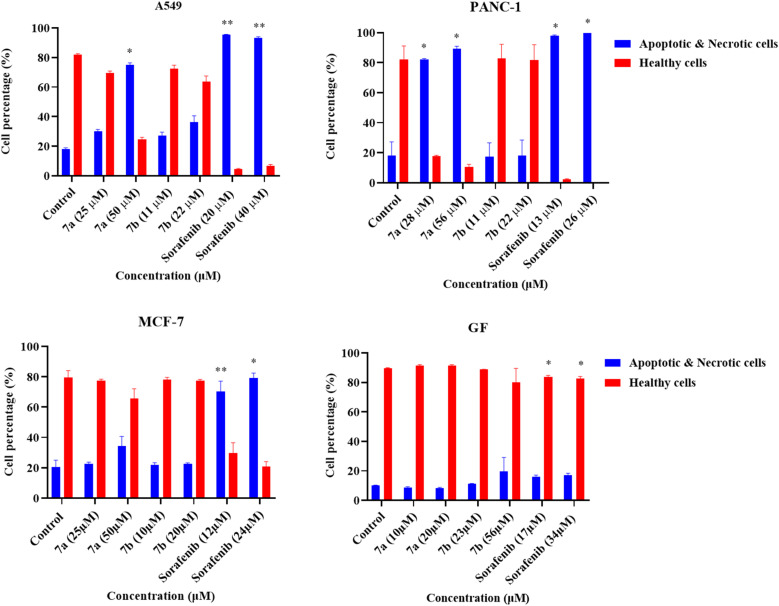
Flow cytometry analysis of the percentages of apoptotic and necrotic cells, and healthy cells treated with IC_50_ and double the IC_50_ of compound 7a, 7b, and sorafenib as a positive control, A549, PANC-1, MCF-7, and GF for 72 hours. Statistical analysis was performed using two-way analysis of variance (ANOVA) with Dunnett's multiple comparisons test. Values are in triplicate and are stated as mean ± standard deviation (SD). *p ≤ 0.05, **p ≤ 0.005, ***p ≤ 0.0005, and ****p ≤ 0.00005 compared to the untreated control.

To determine the cell death modalities, an apoptosis/necrosis assay was conducted. Interestingly, there was a significant increase in the percentages of apoptotic and necrotic cells of A549 cells treated with 50 µM (*p* < 0.05) of compound 7a, 20 µM (*p* < 0.005), and 40 µM (*p* < 0.005) of sorafenib. For the PANC-1 cell line, a significant (*p* < 0.05) increase in the percentages of apoptotic and necrotic cells was observed for cells treated with 28 µM, 56 µM of compound 7a, 13 µM, and 26 µM of sorafenib. A significant elevation in the apoptotic and necrotic cell percentages was recorded for MCF-7 cells treated with 12 µM (*p* < 0.005) and 24 µM (*p* < 0.05), and a significant reduction in the percentages of healthy GF cells treated with 17 µM (*p* < 0.05) and 34 µM (*p* < 0.05) of Sorafenib, as shown in Fig. 7 and S2.

### 3.3 Acridine orange staining (autophagy)

**Fig. 8:**
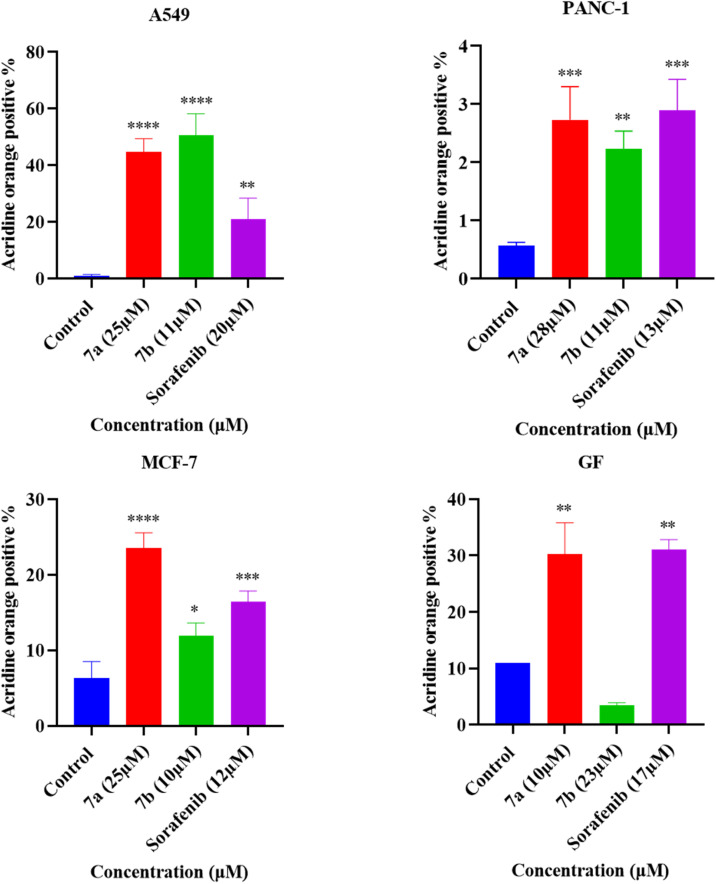
Acridine orange (AO) hydrochloride hydrate staining and percentage of AO-positive A549, PANC-1, MCF-7, and GF cell lines treated with IC_50_ and double the IC_50_ of compound 7a, 7b, and Sorafenib for 72 hours. Statistical analysis was performed by one-way analysis of variance (ANOVA) with Dunnett's multiple comparisons test. Values are in triplicate and are stated as mean ± standard deviation (SD). *p ≤ 0.05, **p ≤ 0.005, ***p ≤ 0.0005, and ****p ≤ 0.00005 compared to the untreated control.

To investigate whether treating A549, PANC-1, MCF-7, and GF cell lines with 7a, 7b, and Sorafenib would lead to the induction of autophagy, acridine orange (AO) stain was used. There was a significant increase in the percentages of AO +ve cells for A549 cells treated with 25 µM of compound 7a, 11 µM of compound 7b, and 20 µM of Sorafenib, in addition, for PANC-1 cells treated with 28 µM of 7a, 11 µM of compound 7b, and 13 µM of Sorafenib. Moreover, a significant increase was observed in 25 µM of compound 7a, 10 µM and 20 µM of compound 7b, and 12 µM of Sorafenib MCF-7 treated cells. For the GF cell line, a significant increase in the percentages AO +ve cells was recorded for 10 µM of the compound 7a, and 17 µM of Sorafenib, as shown in Fig. 8.

### 3.4 Cell cycle analysis

**Fig. 9:**
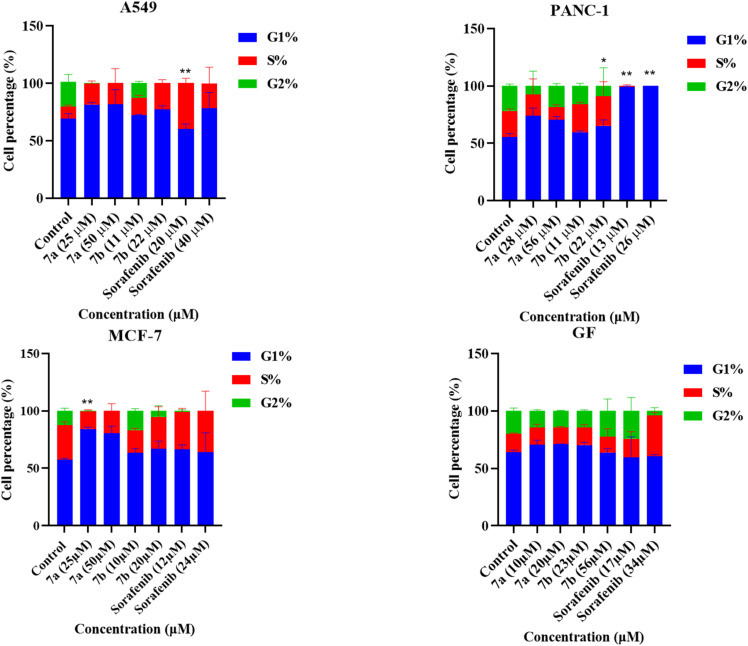
Flow cytometry analysis of cell cycle on PANC-1, A549, MCF-7, and GF treated with IC_50_ and double the IC_50_of compound 7a, 7b, and Sorafenib for 72 hours. Statistical analysis was performed using two-way analysis of variance (ANOVA) with Dunnett's multiple comparisons test. Values are in triplicate and stated as mean ± standard deviation (SD). *p ≤ 0.05, **p ≤ 0.005, ***p ≤ 0.0005, and ****p ≤ 0.00005 compared to the untreated control.

Cell cycle analysis was performed to determine the effect of 7a, 7b, and Sorafenib on cancer cell lines. Regarding PANC-1 cell line, there was a significant arrest in the S-phase when cells were treated with 22 µM of compound 7b and a G1-phase arrest when treated with 13 µM and 26 µM of Sorafenib. Moreover, an S-phase arrest was observed when A549 cells were treated with 20 µM of Sorafenib. In addition, a G1-phase arrest was observed in MCF-7 cells when treated with 25 µM of compound 7a. As shown in Fig. 9 and S2. The results revealed a context-dependent mechanism of cell cycle arrest across different cancer lines, specifically demonstrating both G-1and S phase arrest.

## 4 Discussion

Various quinoline-derived pharmacological agents have received FDA approval for oncological therapies. These derivatives have the capacity to trigger programmed cell death, suppress cellular proliferation, disrupt intracellular signaling cascades, and engage with DNA-associated mechanisms in cancer cells, consequently resulting in the inhibition of growth and the induction of cell death.^12,13^ In this study, novel derivatives of 4-amino-6-phenoxyquinoline and 4,6-diphenoxyqyinoline were synthesized, and their biological effect were screened across breast (MCF-7, MDA-MB-231), glioblastoma (U87), lung (A549), pancreatic (PANC-1), and liver (Hep-G2) cancer cell lines, showing a promising cytotoxic potential.

In the 4-amino-6-phenoxyquinoline derivatives 7a–7c, it was found that compounds 7a and 7b, which have a trifluoromethyl (CF_3_) group at the amine moiety at position 4, exhibited higher potency than 7c, which have benzyl alcohol group. The introduction of a trifluoromethyl (CF_3_) constitutes a significant advancement in the fields of medicinal chemistry and pharmaceutical research. It enables researchers to meticulously adjust the characteristics of organic compounds to maximize their therapeutic efficacy. The integration of a (CF_3_) group within an organic compound can indeed exert a substantial influence on its physicochemical and biological attributes, including lipophilicity, binding affinity, metabolic resilience, and bioavailability.^14,15^ On the other hand, the 4-amino-6-phenoxy quinoline derivatives 7a and 7b exhibited higher potency than 4,6-diphenoxyquinoline 8, which indicates that the amino group at position 4 is more favorable than the phenoxy group.

Notably, compound 7b was approximately as potent as Sorafenib in PANC-1 and MCF-7, and approximately twice as potent as Sorafenib against A549 cells. However, Sorafenib has reduced the percentages of healthy normal GF cells, while compound 7a and compound 7b did not have such an effect on GF cells. The results of this investigation indicate that compounds 7a and 7b exhibit a wide-ranging anticancer efficacy similar to that of sorafenib, while demonstrating increased potency in specific oncological models.^16,17^

In our study, the compound 7a showed a significant increase in the percentages of apoptotic and necrotic cells for A549 and PANC-1 cell lines. This was also reported by a study on 6-cinnamamido-quinoline-4-carboxamide derivatives, which reported an induction of apoptosis through the cleavage of caspase-9 and poly (ADP-ribose) polymerase (PARP).^18^ A quinoline derivative of combretastatin also induced apoptosis,^16^ in addition to the induction of apoptosis/necrosis cell death by a quinolone-based analogue named KA7, synthesized by our research group.^19^

There was a notable induction of autophagy in this study when A549, PANC-1, and MCF-7 were treated with compounds 7a and 7b. This was also reported by a study testing 6-cinnamamido-quinoline-4-carboxamide derivatives on leukemia cell lines through the upregulation of LC3-II.^18^ Moreover, KA7 has induced autophagy in A549 and MCF-7 cell lines.^19^ A study reported that morpholino-4-anilinoquinoline derivative can cause G0G1 arrest in HepG2 cell line,^11^ similar to the results of this study. This could be attributed to interference with the interaction between cyclin-dependent kinase 2 (CDK2) and its associated cyclin A subunit, and through the interruption of cyclin binding is attributable to the conformational alteration occurring within the pivotal C-Helix.^20^

In conclusion, the compounds have a cytotoxic effect against various cancerous cell lines, through initiating autophagy, apoptosis, and inducing cell cycle arrest, indicating that 4-amino-6-phenoxyquinoline derivatives represent a promising therapeutic avenue for oncology.

## Author contributions

Ahmed Alsheikh: conceptualization, data curation, formal analysis, writing – review& editing, funding acquisition. Mohammad Abuoun: data curation, data analysis, writing – review and editing. Hana'a Khalaf: data curation, writing – review and editing. Nour Albaz: data curation, writing – review & editing, Amna Zaytoun: data curation, writing – review& editing. Duaa Abuarqoub: conceptualization, supervision, writing – review & editing.

## Conflicts of interest

The authors declare no conflicts of interest.

## Supplementary Material

RA-016-D6RA01045H-s001

RA-016-D6RA01045H-s002

## Data Availability

All data generated or analyzed during this study are included in this published article and its supplementary information (SI). Supplementary information is available. See DOI: https://doi.org/10.1039/d6ra01045h.
