# Supplementary material for: Design, synthesis, and in vitro antiproliferative activity of novel 4-amino-6-phenoxyquinoline and 4,6-diphenoxyquinoline
Source: RSC Adv. 2026 Apr 30;16(25):22647–58. doi: 10.1039/d6ra01045h (PMC13131335; doi:10.1039/d6ra01045h)
Supplement: RA-016-D6RA01045H-s001 [file RA-016-D6RA01045H-s001.pdf]

Supplementary Information (SI) for RSC Advances.
This journal is © The Royal Society of Chemistry 2026

Figure S1: H-NMR

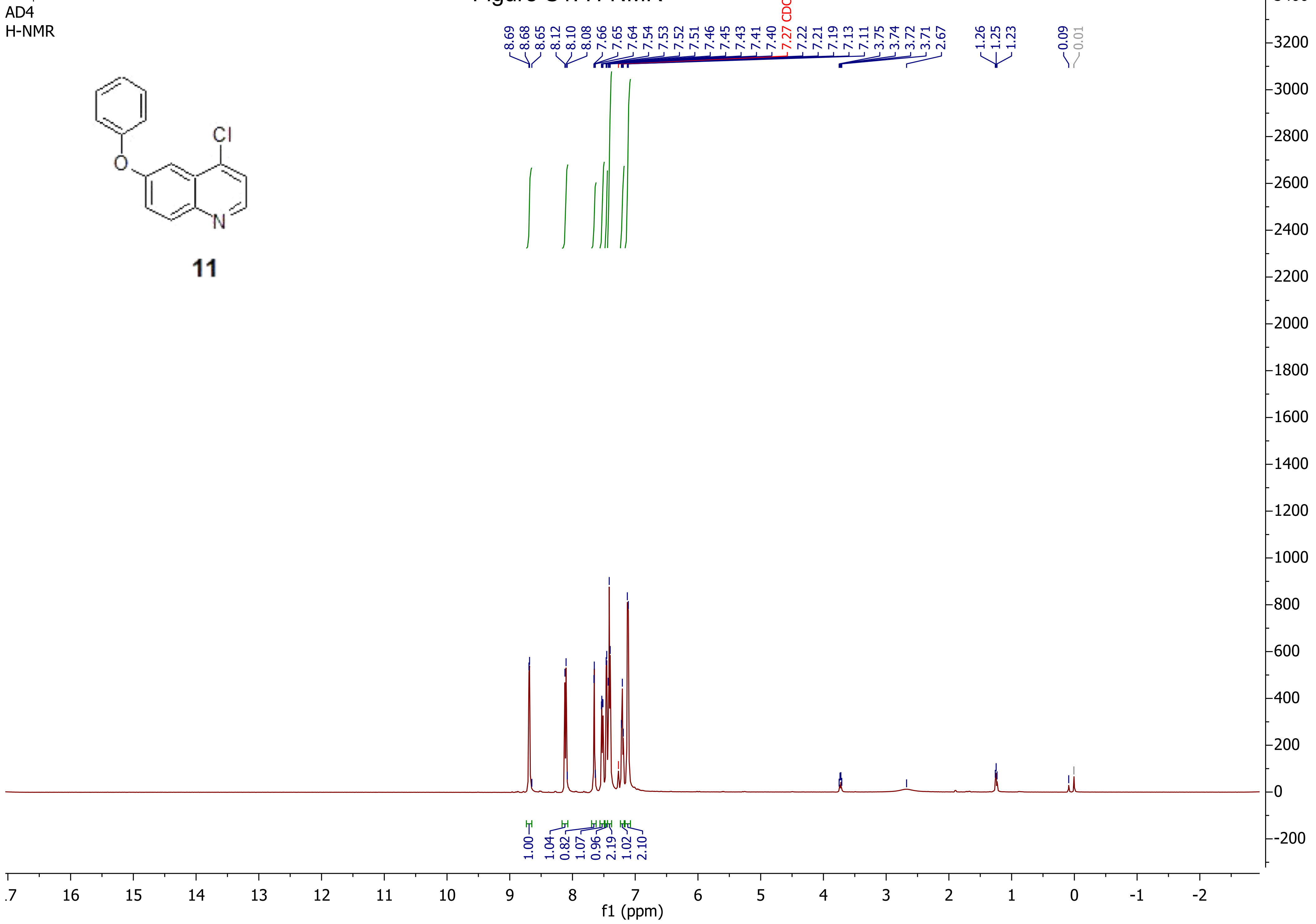

Rar$DRa1352.7287.471.fid
AD6
H-NMR

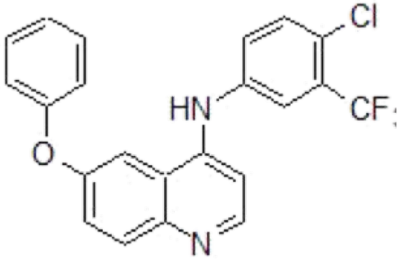

7a

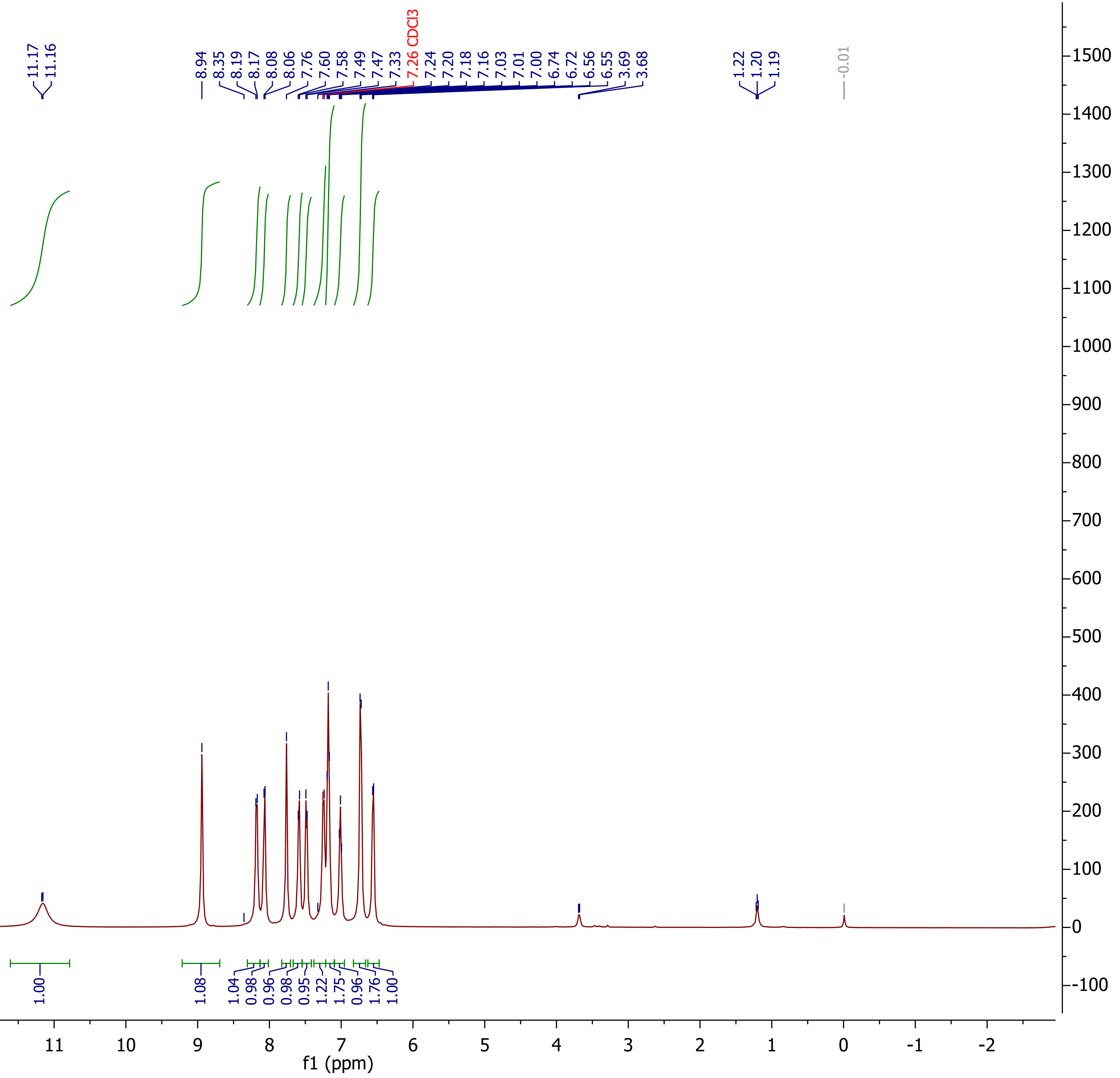

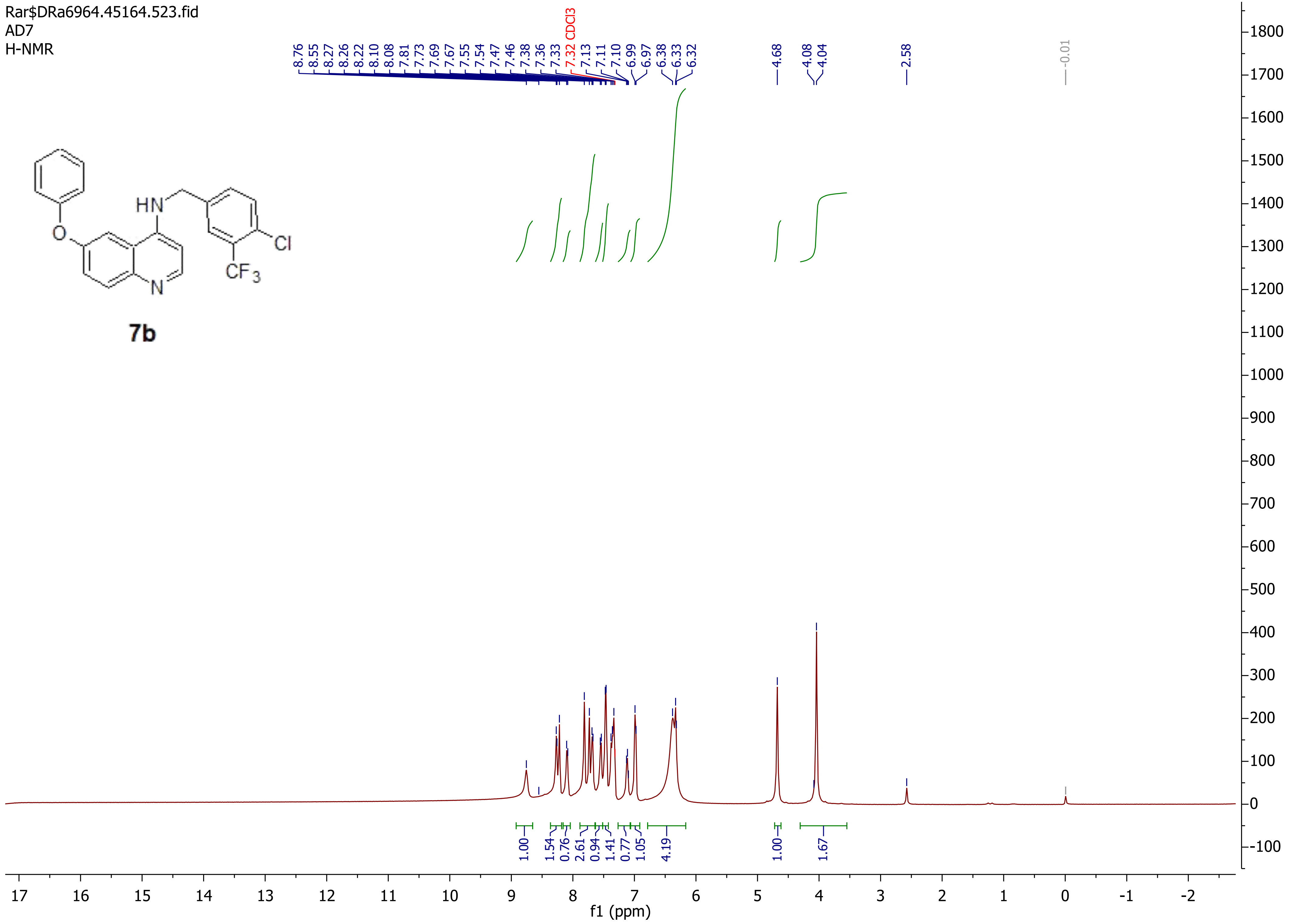
Rar$DRa6964.45164.523.fid
AD7
H-NMR
8.76 8.55 8.27 8.26 8.22 8.10 8.08 7.81 7.73 7.69 7.67 7.55 7.54 7.47 7.46 7.38 7.36 7.33 7.32 CDCl3 7.13 7.11 7.10 6.99 6.97 6.38 6.33 6.32
4.68 4.08 4.04 2.58 -0.01
HN
O
Cl
CF3
N
7b
1.00 1.54 0.76 2.61 0.94 1.41 0.77 1.05 4.19 1.00 1.67
f1 (ppm)

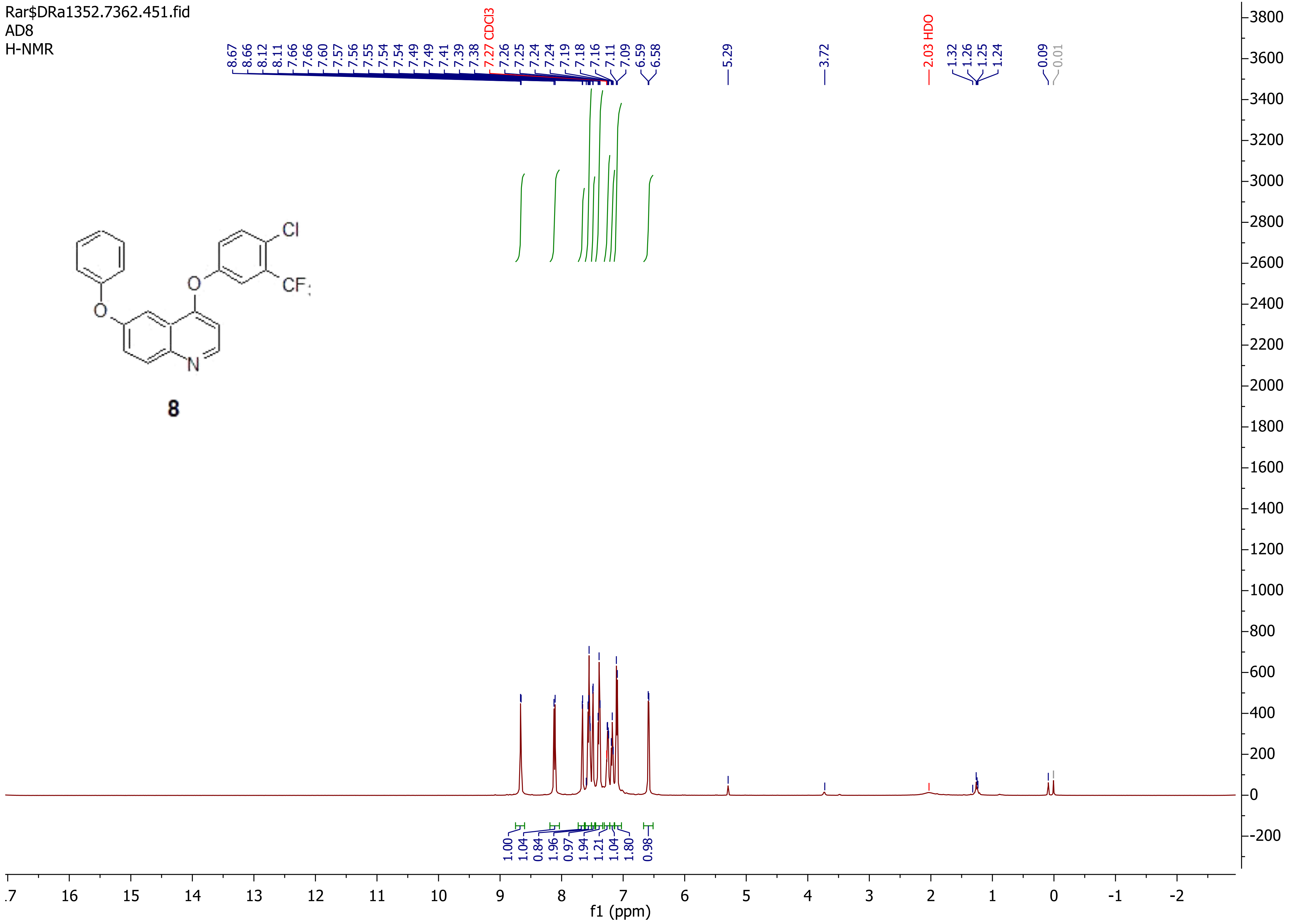
Rar$DRa1352.7362.451.fid
AD8
H-NMR
8.67 8.66 8.12 8.11 7.66 7.66 7.60 7.57 7.56 7.55 7.54 7.54 7.49 7.49 7.41 7.39 7.38
7.27 CDCl3
7.26 7.25 7.24 7.24 7.19 7.18 7.16 7.11 7.09 6.59 6.58
5.29
3.72
2.03 HDO
1.32 1.26 1.25 1.24
0.09 0.01
1.00 1.04 0.84 1.96 0.97 1.94 1.21 1.04 1.80 0.98
f1 (ppm)
Cl
O
CF3
O
N

8

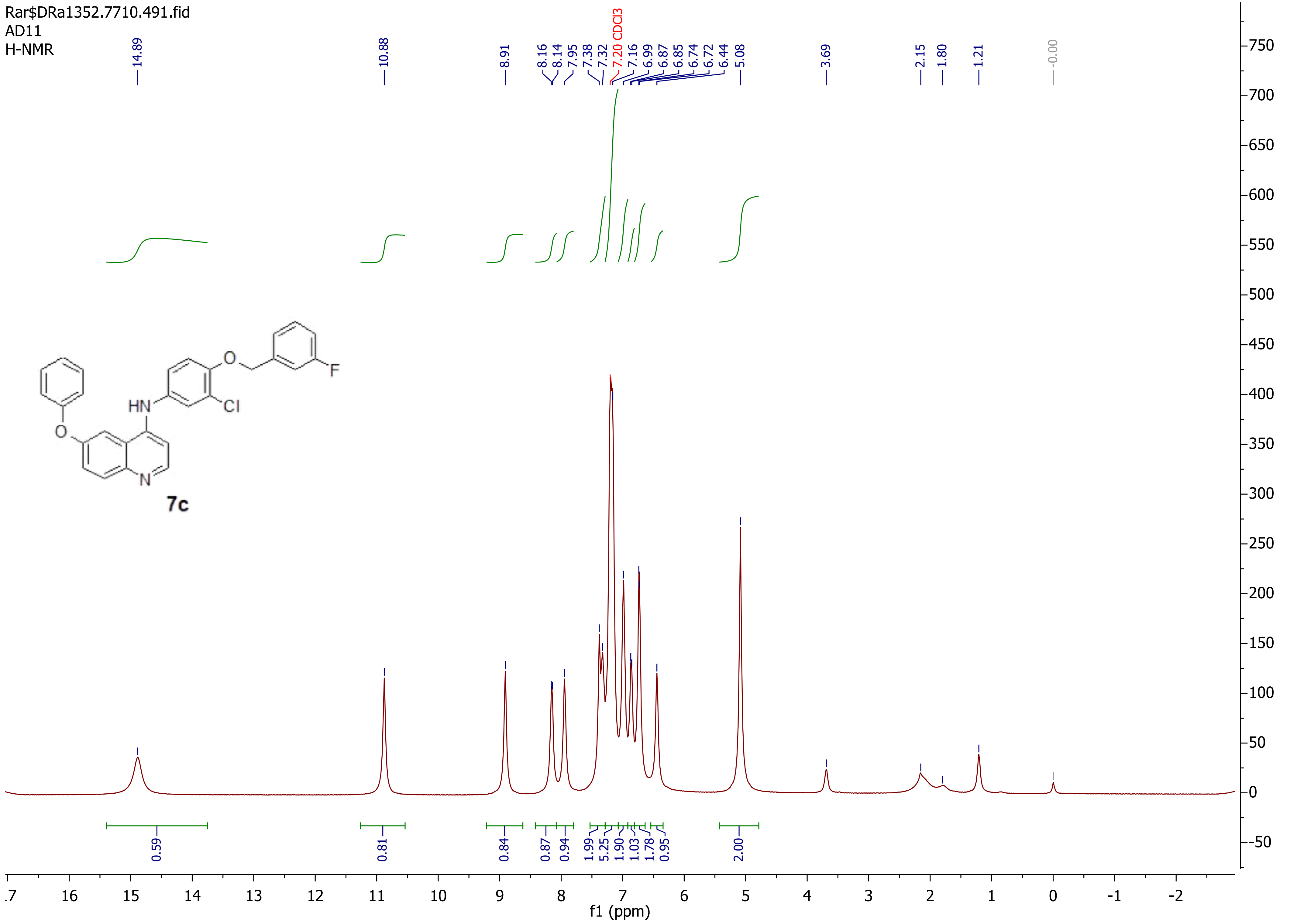
Rar$DRa1352.7710.491.fid
AD11
H-NMR
14.89
10.88
8.91
8.16
8.14
7.95
7.38
7.32
7.20 CDCl3
7.16
6.99
6.87
6.85
6.74
6.72
6.44
5.08
3.69
2.15
1.80
1.21
-0.00
0.59
0.81
0.84
0.87
0.94
1.99
5.25
1.90
1.03
1.78
0.95
2.00
HN
O
O
F
Cl
N
7c
f1 (ppm)

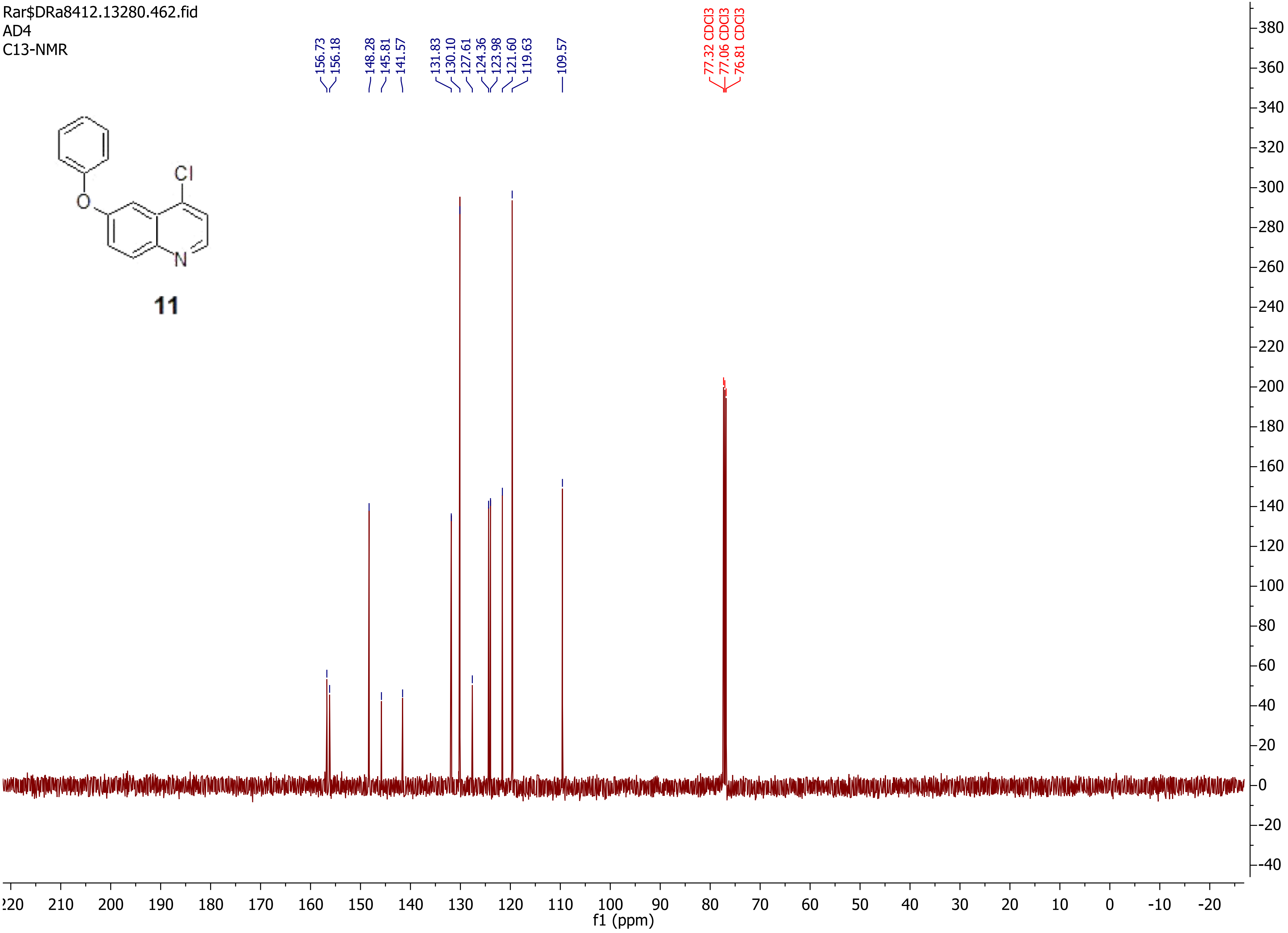
Rar$DRa8412.13280.462.fid
AD4
C13-NMR
156.73
156.18
148.28
145.81
141.57
131.83
130.10
127.61
124.36
123.98
121.60
119.63
109.57
77.32 CDCl3
77.06 CDCl3
76.81 CDCl3
Cl
O
N
11
f1 (ppm)

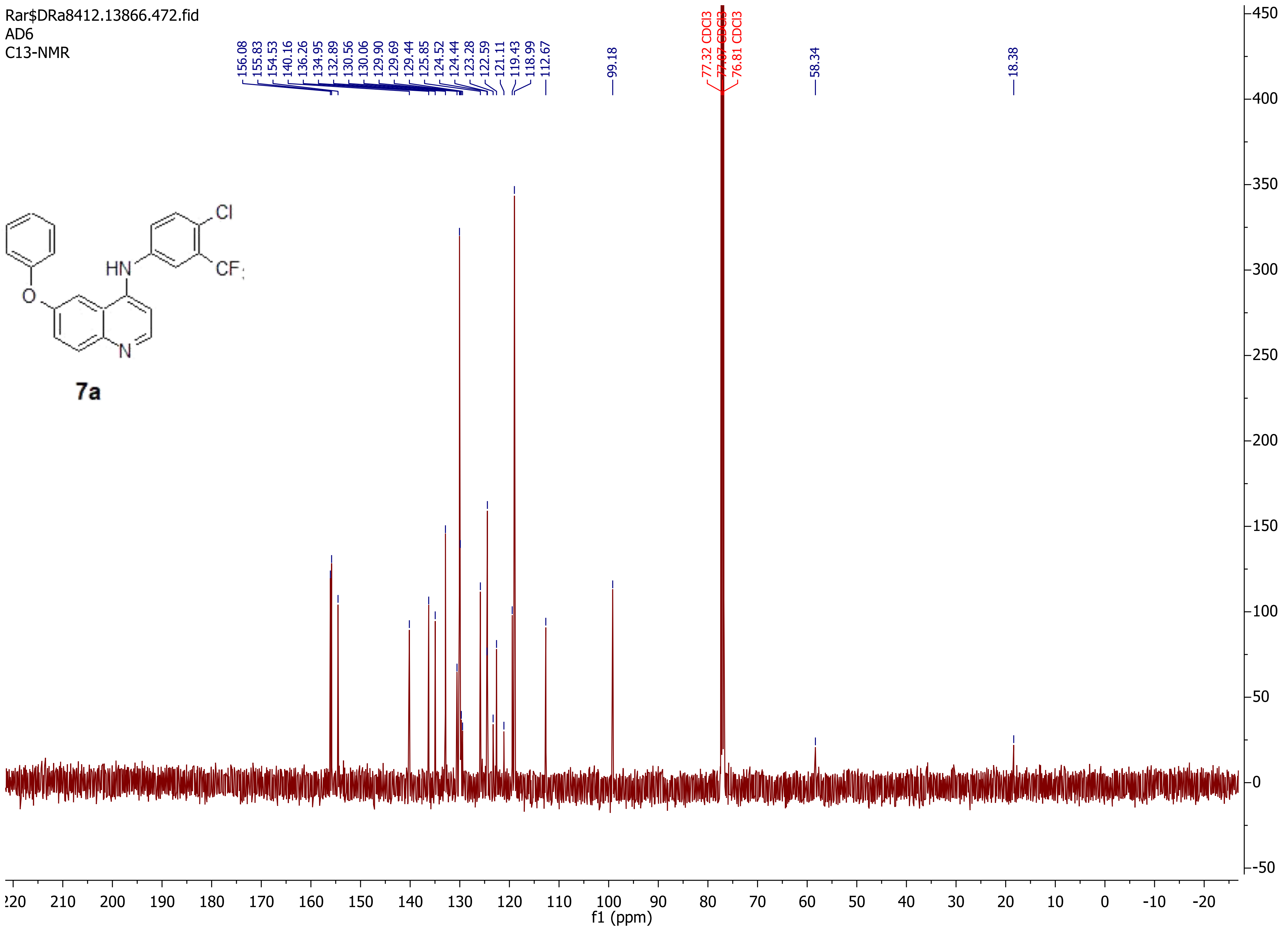
Rar$DRa8412.13866.472.fid
AD6
C13-NMR
156.08
155.83
154.53
140.16
136.26
134.95
132.89
130.56
130.06
129.90
129.69
129.44
125.85
124.52
124.44
123.28
122.59
121.11
119.43
118.99
112.67
99.18
77.32 CDCl3
77.07 CDCl3
76.81 CDCl3
58.34
18.38
Cl
HN
CF
O
N
7a
f1 (ppm)

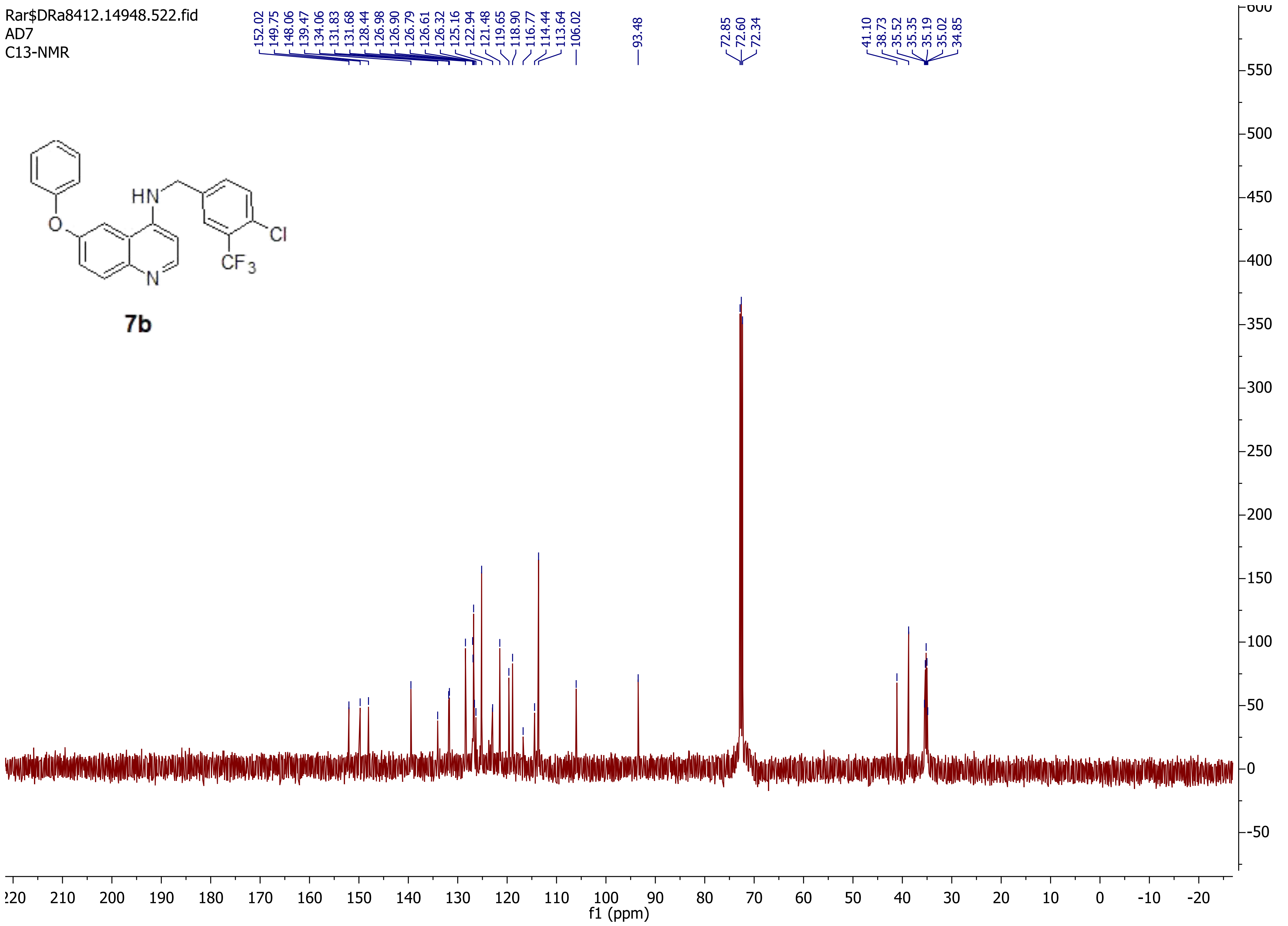
Rar$DRa8412.14948.522.fid
AD7
C13-NMR
152.02 149.75 148.06 139.47 134.06 131.83 131.68 128.44 126.98 126.90 126.79 126.61 126.32 125.16 122.94 121.48 119.65 118.90 116.77 114.44 113.64 106.02
93.48
72.85 72.60 72.34
41.10 38.73 35.52 35.35 35.19 35.02 34.85
HN
O
N
Cl
CF3
7b
f1 (ppm)

Rar$DRa8412.12660.452.fid
AD8
C13-NMR

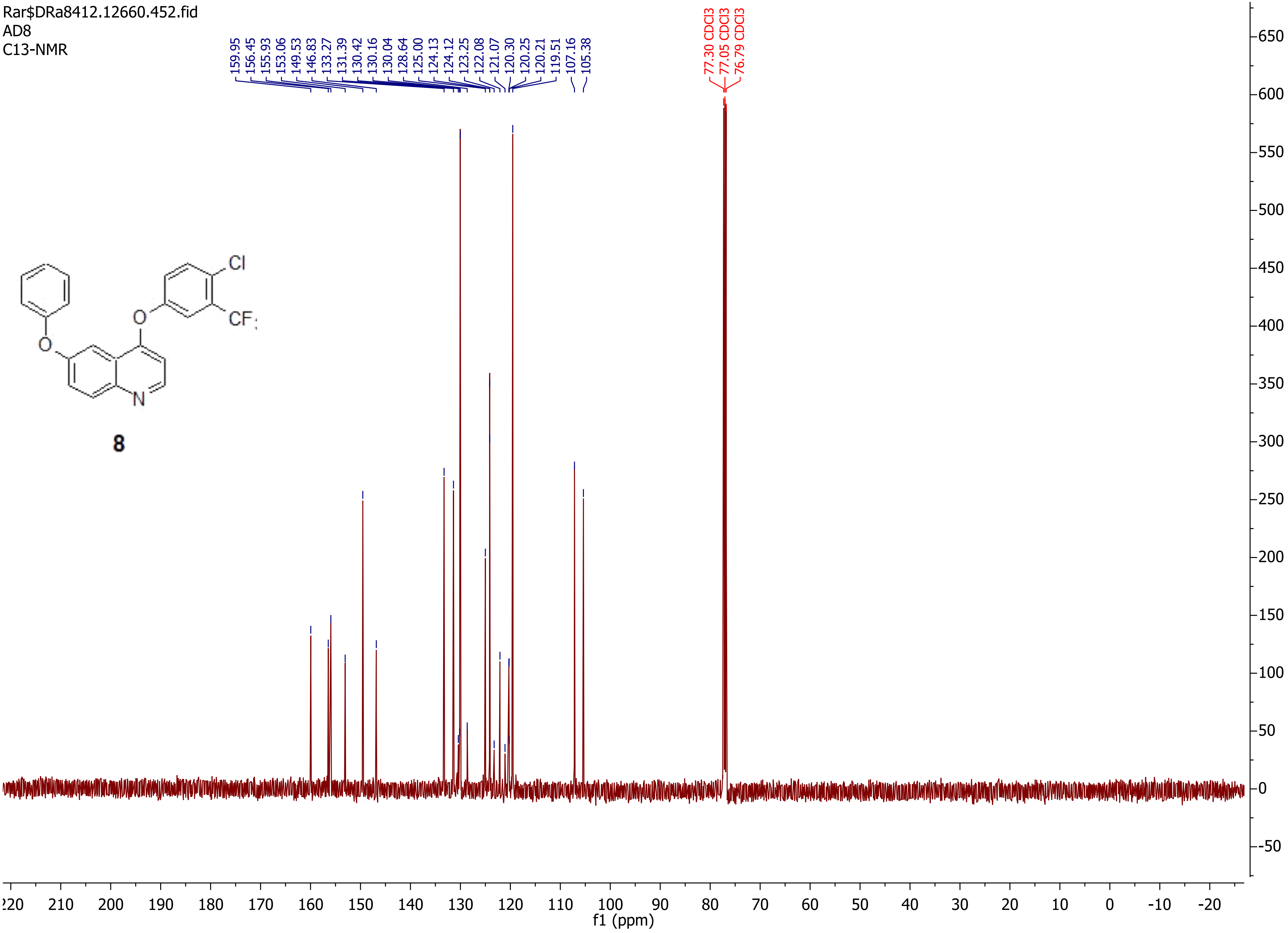

8

Rar$DRa8412.14294.492.fid
AD11
C13-NMR

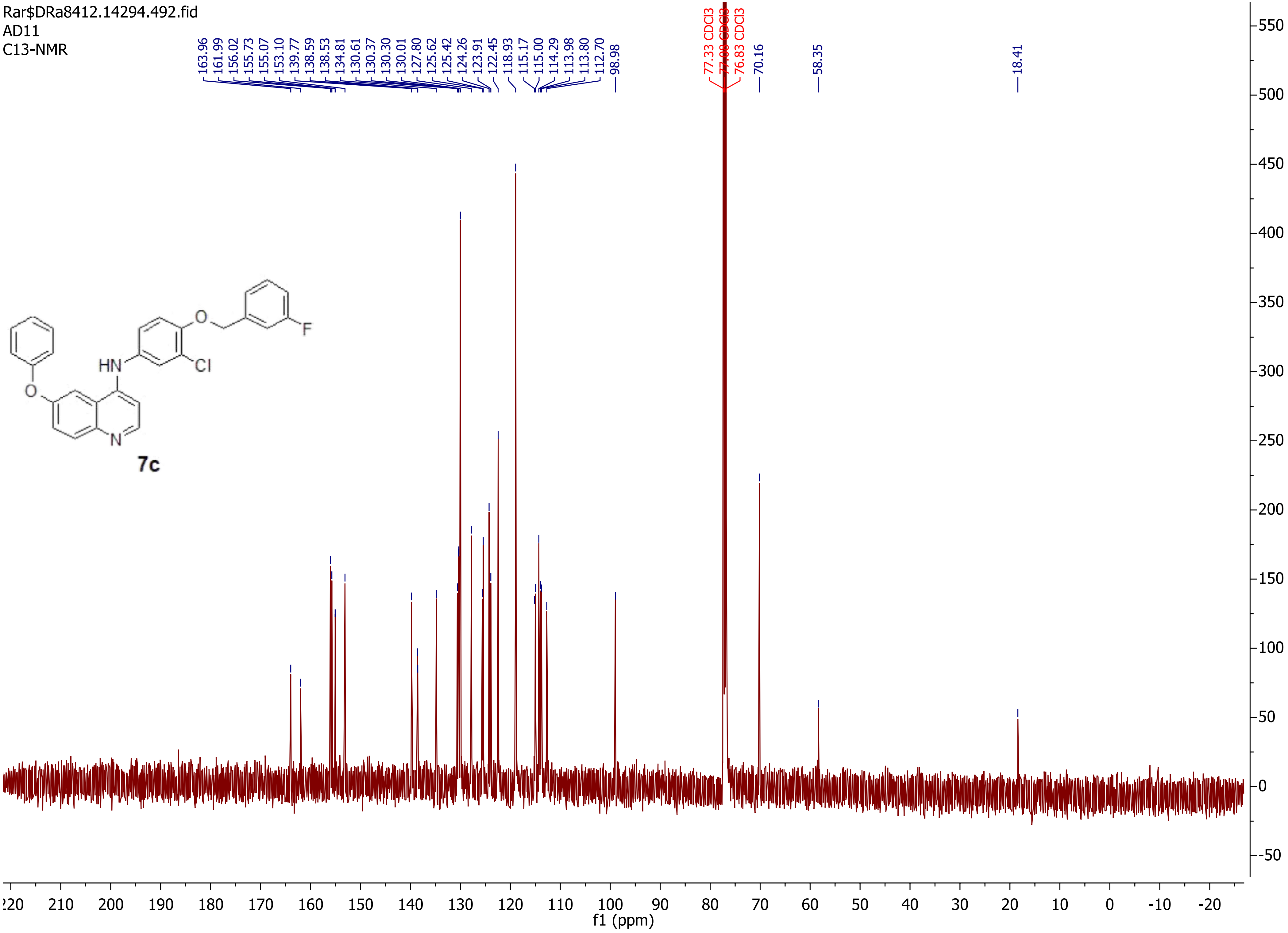

Compound Spectrum List Report

**Analysis Info**	QAD 4	Acquisition Date	9/09/2023
Method	Kailani_MS/MS	Operator	Naba Hikma
		Instrument impact II	1825265.10265

Acquisition Parameter

Source Type	ESI	Ion Polarity	Positive	Set Nebulizer	2.0 Bar
Focus	Active	Set Capillary	2500 V	Set Dry Heater	200 °C
Scan Begin	30 m/z	Set End Plate Offset	-500 V	Set Dry Gas	8.0 l/min
Scan End	1000 m/z	Set Charging Voltage	2000 V		

C15H10ClNO

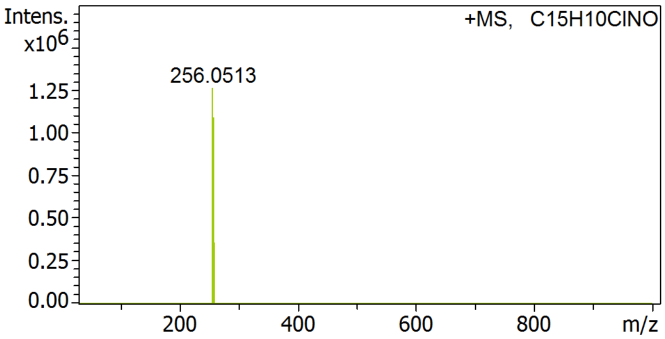

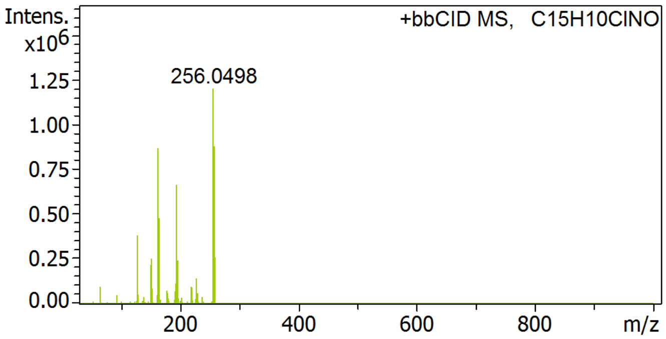

#	m/z	Res.	S/N	I	I %	FWHM
1	255.9523	13218	3.8	5269	0.4	0.0194
2	255.9924	8258	4.7	6453	0.5	0.0310
3	256.0513	11142	910.2	1262725	100.0	0.0230
4	256.1720	48651	22.1	30628	2.4	0.0053
5	256.2640	33506	12.2	16961	1.3	0.0076
6	256.5444	45370	61.1	84718	6.7	0.0057
7	257.0524	26109	637.6	884470	70.0	0.0098
8	258.0474	17565	784.1	1087719	86.1	0.0147
9	259.0493	37389	258.7	358946	28.4	0.0069
10	260.0524	34339	21.7	30166	2.4	0.0076

#	m/z	Res.	S/N	I	I %	FWHM
1	128.0474	27357	311.7	381534	31.7	0.0047
2	151.0158	28006	208.0	254679	21.2	0.0054
3	163.0162	16987	709.4	868495	72.1	0.0096
4	165.0128	28454	392.5	480484	39.9	0.0058
5	195.0053	28421	541.8	663260	55.1	0.0069
6	197.0023	32122	198.4	242864	20.2	0.0061
7	256.0498	13465	983.4	1203855	100.0	0.0190
8	257.0523	27456	526.4	644439	53.5	0.0094
9	258.0460	20512	717.5	878421	73.0	0.0126
10	259.0493	38016	213.5	261402	21.7	0.0068

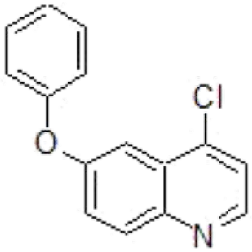

11

Compound Spectrum List Report

**Analysis Info**	QAD 6	Acquisition Date	9/09/2023
Method	Kailani_MS/MS	Operator	Naba Hikma
		Instrument impact II	1825265.10265

Acquisition Parameter

Source Type	ESI	Ion Polarity	Positive	Set Nebulizer	2.0 Bar
Focus	Active	Set Capillary	2500 V	Set Dry Heater	200 °C
Scan Begin	30 m/z	Set End Plate Offset	-500 V	Set Dry Gas	8.0 l/min
Scan End	1000 m/z	Set Charging Voltage	2000 V		

C22H14F3ClN2O

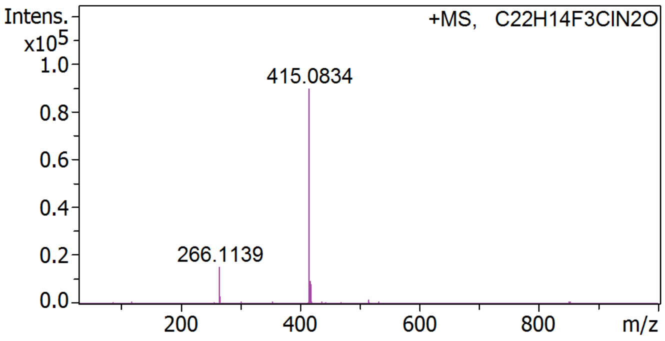

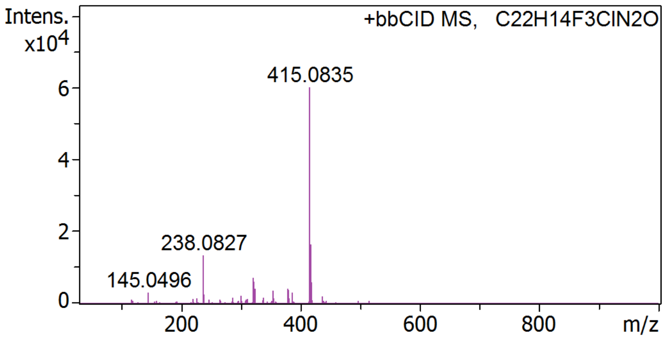

#	m/z	Res.	S/N	I	I %	FWHM
1	266.1139	34089	169.9	15521	17.3	0.0078
2	267.1173	30505	32.8	2995	3.3	0.0088
3	301.9958	34065	11.6	1063	1.2	0.0089
4	414.9764	14414	11.8	1081	1.2	0.0288
5	415.0834	11656	983.5	89837	100.0	0.0356
6	418.0767	45196	106.4	9720	10.8	0.0093
7	419.0799	42664	91.9	8391	9.3	0.0098
8	420.0832	36013	10.4	951	1.1	0.0117
9	515.1636	39021	17.5	1599	1.8	0.0132
10	851.1284	42273	11.5	1050	1.2	0.0201

#	m/z	Res.	S/N	I	I %	FWHM
1	238.0827	32448	223.9	13495	22.4	0.0073
2	321.0352	33409	123.4	7442	12.4	0.0096
3	322.0427	34866	105.6	6364	10.6	0.0092
4	324.0399	31348	73.1	4407	7.3	0.0103
5	354.0328	33063	63.2	3809	6.3	0.0107
6	379.0995	35585	73.4	4423	7.4	0.0107
7	380.1058	31392	67.3	4057	6.7	0.0121
8	415.0835	11096	997.5	60135	100.0	0.0374
9	418.0765	45010	275.8	16627	27.6	0.0093
10	419.0796	42586	102.7	6192	10.3	0.0098

Type text here

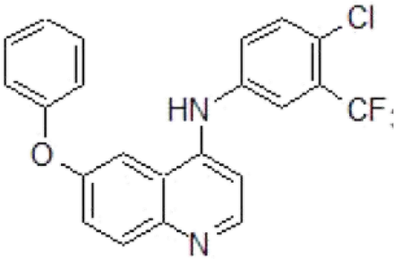

7a

Compound Spectrum List Report

**Analysis Info**	QAD 7	Acquisition Date	9/09/2023
Method	Kailani_MS/MS	Operator	Naba Hikma
		Instrument impact II	1825265.10265

Acquisition Parameter

Source Type	ESI	Ion Polarity	Positive	Set Nebulizer	2.0 Bar
Focus	Active	Set Capillary	2500 V	Set Dry Heater	200 °C
Scan Begin	30 m/z	Set End Plate Offset	-500 V	Set Dry Gas	8.0 l/min
Scan End	1000 m/z	Set Charging Voltage	2000 V		

C23H16F3ClN2O

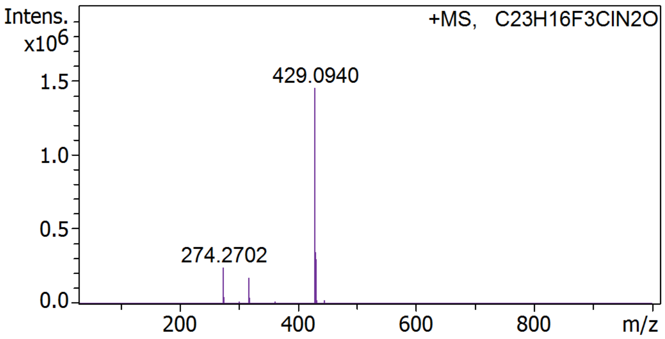

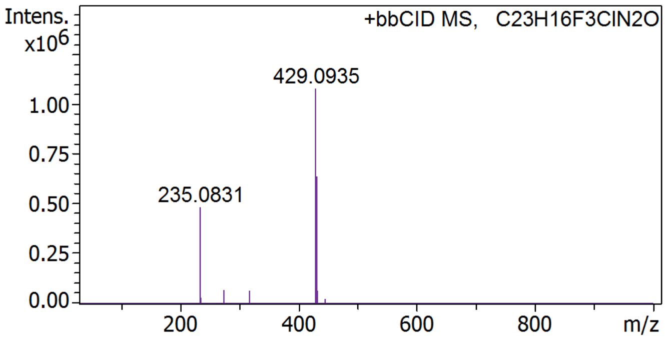

#	m/z	Res.	S/N	I	I %	FWHM
1	274.2702	36172	161.2	247282	17.0	0.0076
2	275.2736	33459	28.8	44200	3.0	0.0082
3	318.2961	37424	115.0	176342	12.1	0.0085
4	319.2995	34928	25.3	38880	2.7	0.0091
5	429.0940	19165	946.2	1451508	100.0	0.0224
6	429.7299	56700	23.6	36128	2.5	0.0076
7	430.0951	36794	227.0	348206	24.0	0.0117
8	431.0894	31350	197.5	302897	20.9	0.0138
9	432.0920	46603	15.9	24459	1.7	0.0093
10	445.0866	38934	14.2	21714	1.5	0.0114

#	m/z	Res.	S/N	I	I %	FWHM
1	235.0831	24910	445.9	485355	44.9	0.0094
2	236.0891	32676	28.0	30525	2.8	0.0072
3	274.2701	35749	66.5	72376	6.7	0.0077
4	318.2960	37770	62.4	67903	6.3	0.0084
5	429.0935	19148	992.1	1079857	100.0	0.0224
6	429.7300	57748	24.6	26727	2.5	0.0074
7	430.0950	36583	392.0	426701	39.5	0.0118
8	431.0892	31558	585.8	637565	59.0	0.0137
9	432.0920	46686	64.3	69967	6.5	0.0093
10	445.0865	39215	20.6	22464	2.1	0.0113

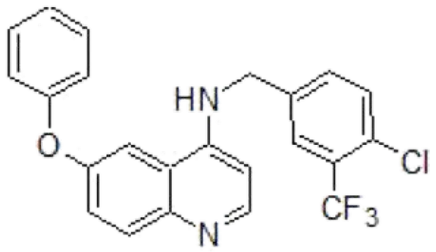

7b

Compound Spectrum List Report

**Analysis Info**	QAD 8	Acquisition Date	9/09/2023
Method	Kailani_MS/MS	Operator	Naba Hikma
		Instrument impact II	1825265.10265

Acquisition Parameter

Source Type	ESI	Ion Polarity	Positive	Set Nebulizer	2.0 Bar
Focus	Active	Set Capillary	2500 V	Set Dry Heater	200 °C
Scan Begin	30 m/z	Set End Plate Offset	-500 V	Set Dry Gas	8.0 l/min
Scan End	1000 m/z	Set Charging Voltage	2000 V		

C22H13F3ClNO2

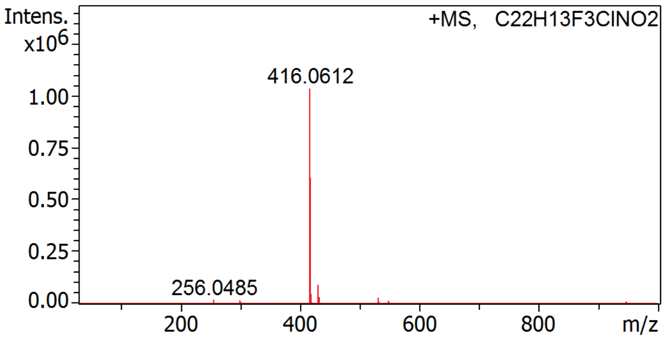

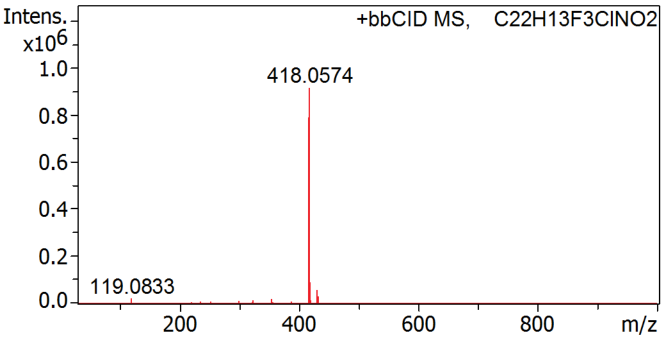

#	m/z	Res.	S/N	I	I %	FWHM
1	256.0485	34866	18.7	19516	1.9	0.0073
2	299.9974	35562	14.8	15404	1.5	0.0084
3	416.0612	23142	994.2	1036211	100.0	0.0180
4	417.0633	47170	258.6	269504	26.0	0.0088
5	418.0572	43528	581.6	606146	58.5	0.0096
6	419.0605	45316	47.5	49512	4.8	0.0092
7	430.0755	41993	89.5	93273	9.0	0.0102
8	431.0790	38481	31.5	32797	3.2	0.0112
9	432.0730	37444	29.7	30948	3.0	0.0115
10	531.2667	41526	26.5	27650	2.7	0.0128

#	m/z	Res.	S/N	I	I %	FWHM
1	119.0833	25123	23.5	21670	2.4	0.0047
2	323.0269	37869	17.5	16136	1.8	0.0085
3	355.0168	37179	21.0	19330	2.1	0.0095
4	416.0615	20361	856.2	789017	86.2	0.0204
5	417.0634	45359	466.4	429794	47.0	0.0092
6	418.0574	38135	993.1	915213	100.0	0.0110
7	419.0606	45862	101.1	93196	10.2	0.0091
8	430.0755	42544	65.3	60187	6.6	0.0101
9	431.0788	39564	29.7	27376	3.0	0.0109
10	432.0729	37809	34.8	32110	3.5	0.0114

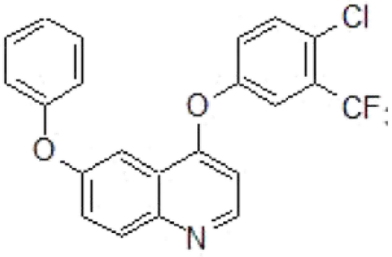

8

Compound Spectrum List Report

**Analysis Info**	QAD 11	Acquisition Date	9/09/2023
Method	Kailani_MS/MS	Operator	Naba Hikma
		Instrument impact II	1825265.10265

Acquisition Parameter

Source Type	ESI	Ion Polarity	Positive	Set Nebulizer	2.0 Bar
Focus	Active	Set Capillary	2500 V	Set Dry Heater	200 °C
Scan Begin	30 m/z	Set End Plate Offset	-500 V	Set Dry Gas	8.0 l/min
Scan End	1000 m/z	Set Charging Voltage	2000 V		

C28H20FClN2O2

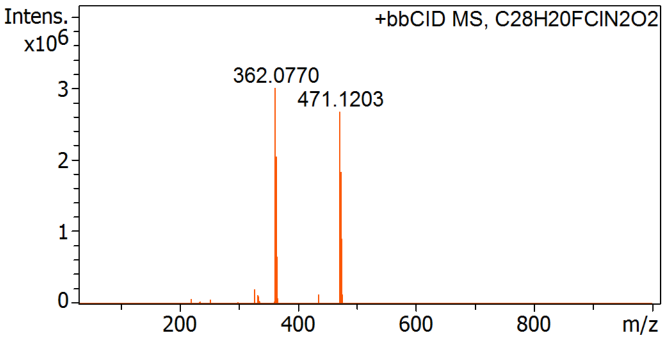

#	m/z	Res.	S/N	I	I %	FWHM
1	327.1077	36821	1277755.3	210678	7.0	0.0089
2	362.0770	19829	18252024.0	3009413	100.0	0.0183
3	363.0798	31022	11087186.0	1828067	60.7	0.0117
4	364.0743	25125	12424277.0	2048528	68.1	0.0145
5	365.0766	39461	3988196.3	657578	21.9	0.0093
6	435.1443	40530	789934.5	130245	4.3	0.0107
7	471.1203	20052	16168759.0	2665922	88.6	0.0235
8	472.1244	27032	10902825.0	1797669	59.7	0.0175
9	473.1185	24229	11141909.0	1837090	61.0	0.0195
10	474.1216	48615	5508679.0	908277	30.2	0.0098

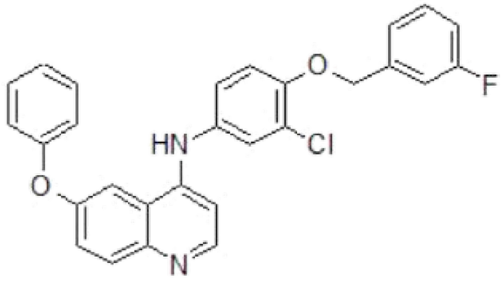

7c

Compound Spectrum List Report

C28H20FClN2O2

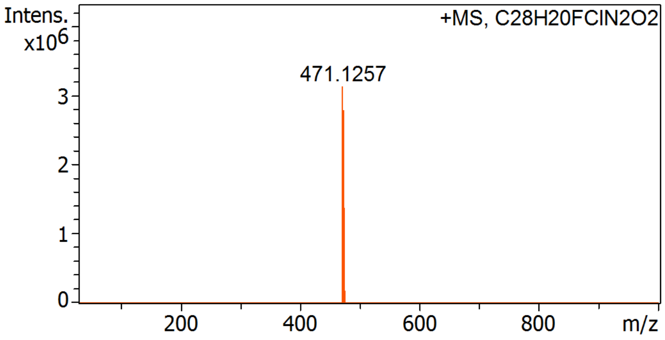

#	m/z	Res.	S/N	I	I %	FWHM
1	471.0115	13923	92330.3	12981	0.4	0.0338
2	471.1257	15153	22255818.0	3128971	100.0	0.0311
3	471.2871	68322	259092.5	36426	1.2	0.0069
4	471.4120	43227	216421.5	30427	1.0	0.0109
5	471.7900	57501	785442.4	110426	3.5	0.0082
6	472.1253	26686	18789296.0	2641609	84.4	0.0177
7	473.1204	23577	19804288.0	2784308	89.0	0.0201
8	474.1216	47332	9783370.0	1375455	44.0	0.0100
9	475.1246	44551	1367273.9	192227	6.1	0.0107
10	476.1277	38222	88705.5	12471	0.4	0.0125
