# Supplementary material for: Design, synthesis, and in vitro antiproliferative activity of novel 4-amino-6-phenoxyquinoline and 4,6-diphenoxyquinoline
Source: RSC Adv. 2026 Apr 30;16(25):22647–58. doi: 10.1039/d6ra01045h (PMC13131335; doi:10.1039/d6ra01045h)
Supplement: RA-016-D6RA01045H-s002 [file RA-016-D6RA01045H-s002.pdf]

This journal is © The Royal Society of Chemistry 2026

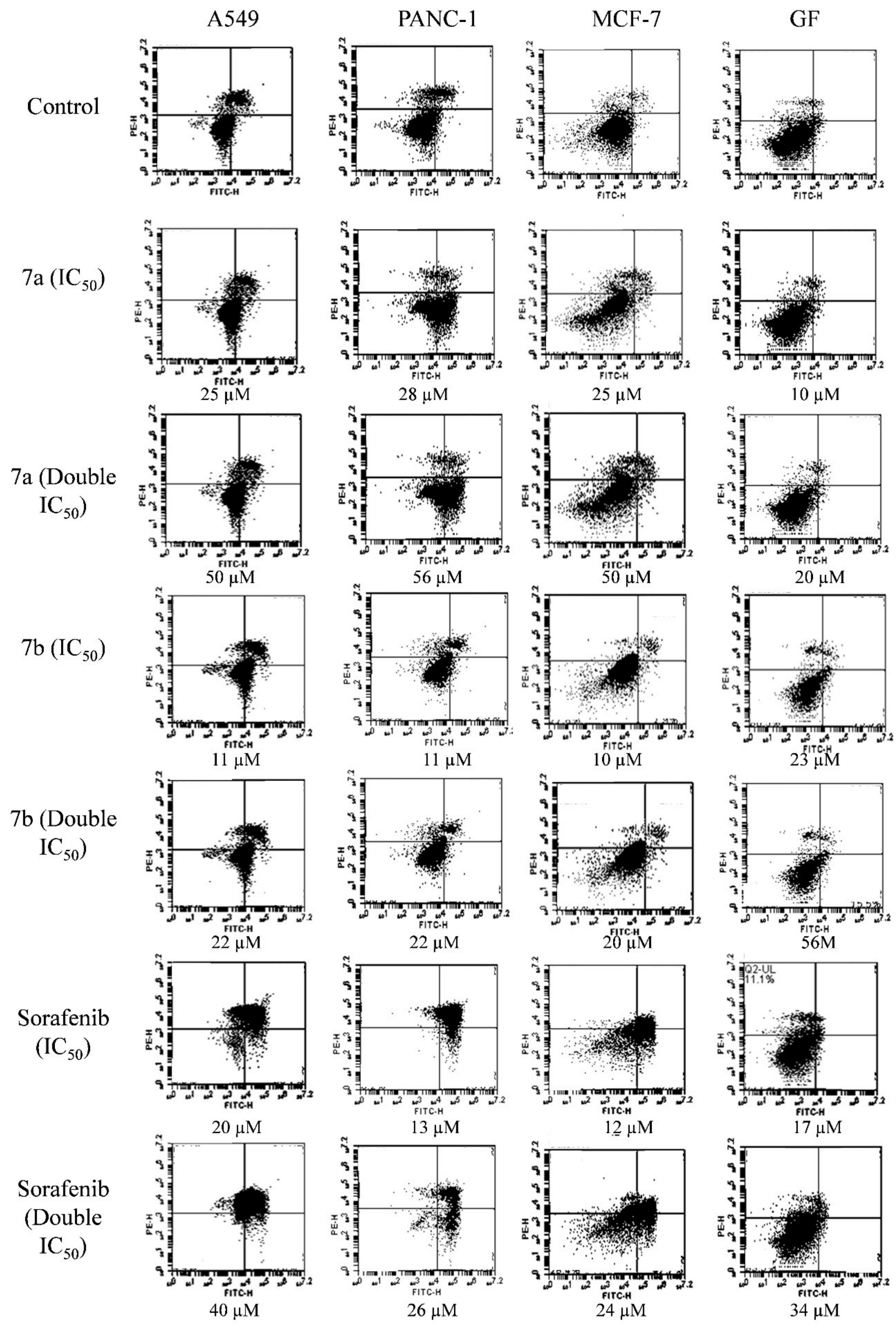

FigS2: Flow cytometry dot plots of A549, PANC-1, MCF-7, and GF cell lines treated with IC_{50} and double the IC_{50} of compound **7a**, **7b**, and Sorafenib for 72 hours compared to their respective negative untreated control. The quadrants (Q) represent the percentages of cells in the following order: Q1-upper left (UL): necrotic, Q1-upper right (UR) and lower right (LR): apoptosis, and Q1-lower left (LL): healthy cells.

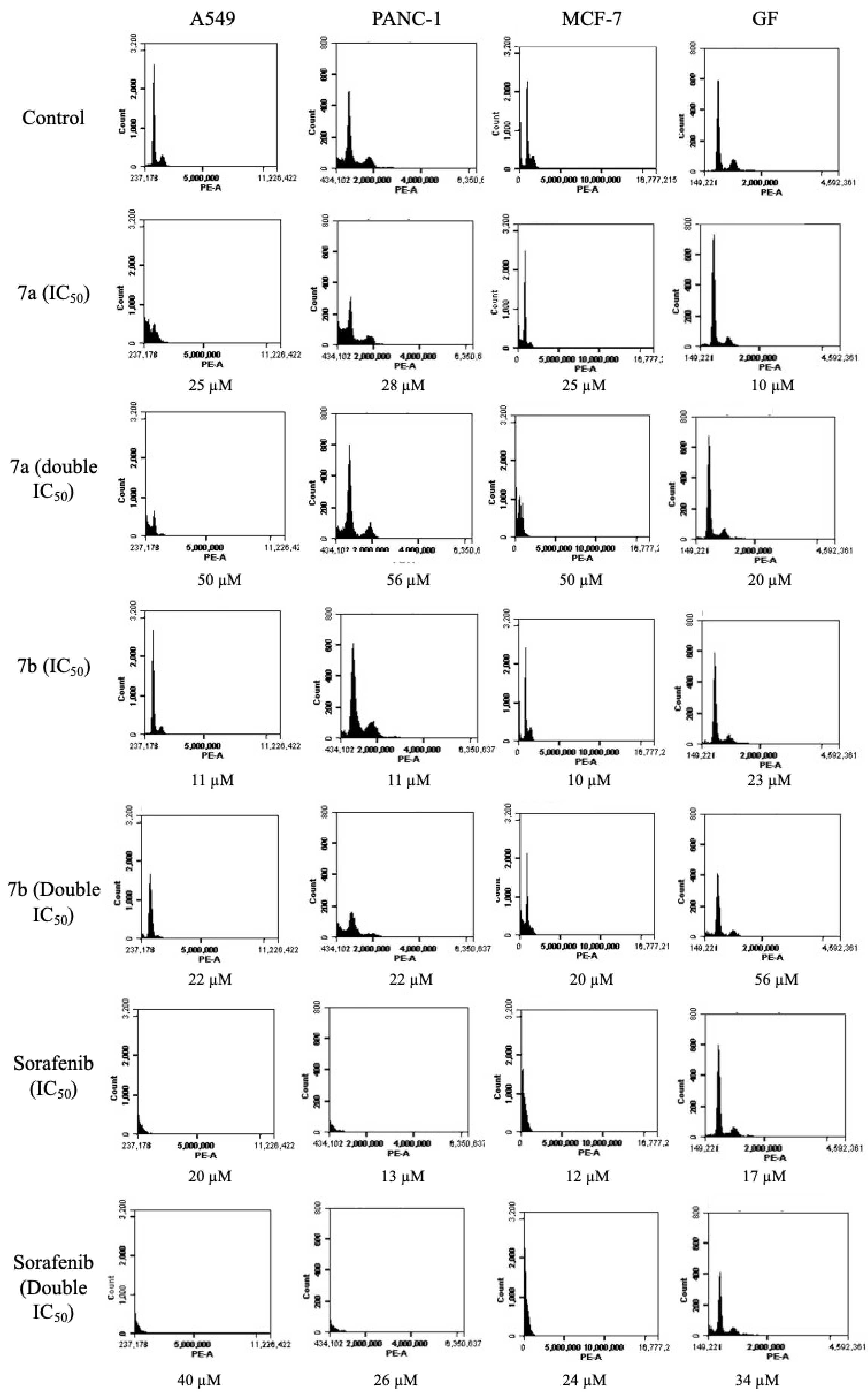

A549
PANC-1
MCF-7
GF
Control
7a (IC_{50})
25 µM
28 µM
25 µM
10 µM
7a (double IC_{50})
50 µM
56 µM
50 µM
20 µM
7b (IC_{50})
11 µM
11 µM
10 µM
23 µM
7b (Double IC_{50})
22 µM
22 µM
20 µM
56 µM
Sorafenib (IC_{50})
20 µM
13 µM
12 µM
17 µM
Sorafenib (Double IC_{50})
40 µM
26 µM
24 µM
34 µM
Count
PE-A

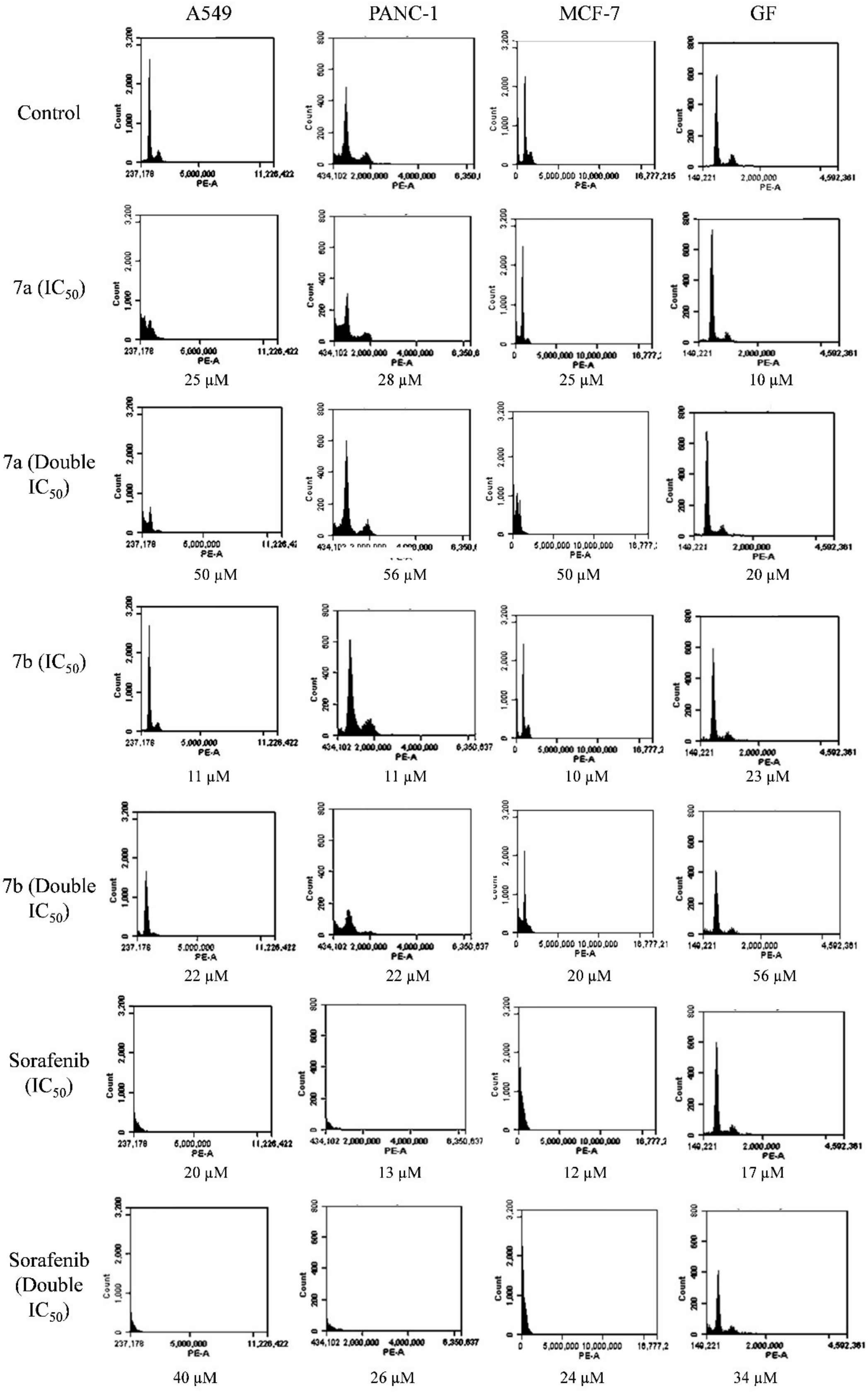
A549
PANC-1
MCF-7
GF
Control
7a (IC_{50})
25 µM
28 µM
25 µM
10 µM
7a (Double IC_{50})
50 µM
56 µM
50 µM
20 µM
7b (IC_{50})
11 µM
11 µM
10 µM
23 µM
7b (Double IC_{50})
22 µM
22 µM
20 µM
56 µM
Sorafenib (IC_{50})
20 µM
13 µM
12 µM
17 µM
Sorafenib (Double IC_{50})
40 µM
26 µM
24 µM
34 µM
Count
PE-A

Fig.S3: Flow cytometry histogram of cell cycle analysis of A549, PANC-1, MCF-7, and GF cell lines treated with IC_{50} and double the IC_{50} of compound **7a**, **7b**, and Sorafenib for 72 hours.
